# Supplementary material for: Compact light field photography towards versatile three-dimensional vision
Source: Nat Commun. 2022 Jun 9;13:3333. doi: 10.1038/s41467-022-31087-9 (PMC9184585; doi:10.1038/s41467-022-31087-9)
Supplement: Supplementary file 1 — Supplementary Information [file 41467_2022_31087_MOESM1_ESM.pdf]

Supplemental Materials for
Compact light field photography towards versatile three-dimensional vision

Xiaohua Feng[1+*], Yayao Ma[2+], Liang Gao[2*]
[1]Research Center for Humanoid Sensing, Zhejiang Laboratory, Hangzhou, 311121, China.
[2]Department of Bioengineering, University of California, Los Angeles, 90064, USA.
[+]These authors contributed equally to this work.
**email: fengxiaohua@zhejianglab.com, gaol@ucla.edu*

Supplementary Note 1. Fundamental assumptions and modeling of CLIP

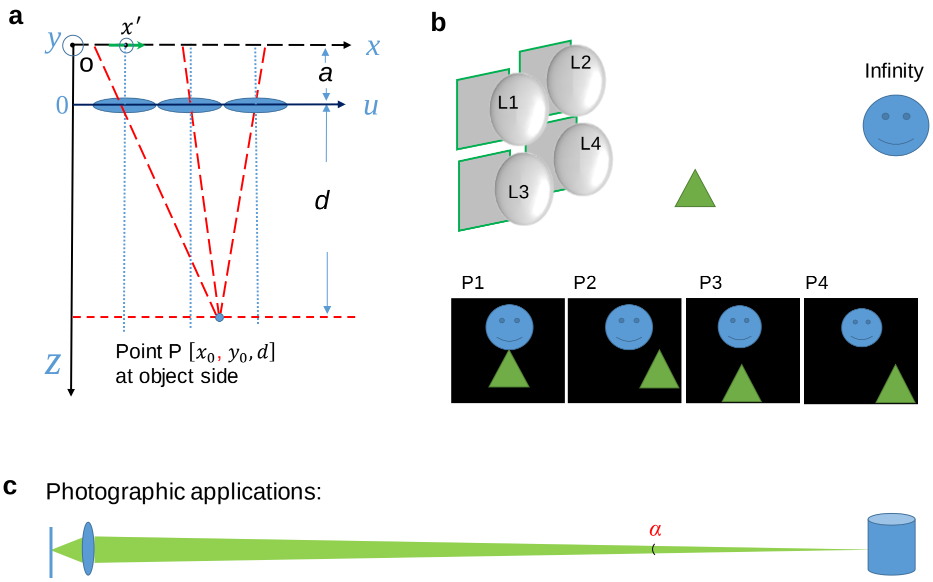

Supplementary Figure 1: CLIP image acquisition. a Two-plane parameterization of the light field. The spatial axis (x) is on the sensor plane, and the angular axis (u) is on the lens' aperture plane. **b** Sub-aperture images (P1-P4) of the same scene from different views L1-L4 with exaggerated image disparities for illustration. Note that the 'smiling face' object is at infinity. Its disparities among different apertures are negligible, whereas the disparities for a close object are much more apparent, illustrating the depth-dependence of the disparity. **c** In photographic applications, the numerical aperture at the object side is typically small, leading to uniform light intensity across the angular range spanned by the lens.

Using the two-plane light field parameterization shown in supplementary Fig. 1a, we established a local coordinate x' (in green) for each sub-aperture image behind the lens and chose the image of a point source at infinity as its origin (indicated by the dashed blue lines). The local image coordinate of a point source at $[x_0, y_0, d]$ in the object space is then:

$$\begin{cases} x' = \frac{a}{d}(u - x_0) & (a) \\ y' = \frac{a}{d}(v - x_0) & (b). \end{cases} \quad (1)$$

Indexing view k in the angular coordinate as (u_k, v_k), the location of a point source in the sub-aperture image can thus be related to that in a reference sub-aperture image via a shear operation in the ray space:

$$\begin{cases} x'_k = \frac{a}{d}(u_k - x_0) = x'_0 + \frac{a}{d}(u_k - u_0) = x'_0 + \frac{a}{d}u_k = x'_0 - su_k & (a) \\ y'_k = \frac{a}{d}(u_k - x_0) = y'_0 + \frac{a}{d}(v_k - v_0) = y'_0 + \frac{a}{d}v_k = y'_0 - sv_k & (b), \end{cases} \quad (2)$$

where $s = -\frac{a}{d}$ is a depth-dependent shearing factor. $u_0 = N_{refx}$ and $v_0 = N_{refy}$ are the indices of the reference sub-aperture, and they are assumed here to be 0 (the central view) for simplicity but can be arbitrary values for viewpoint synthesis. Supplementary Figure 1b shows the depth-dependent disparity between the sub-aperture images.

To relate the sub-aperture images to each other, one also needs to establish the intensity relationships besides the location correspondence for every object point. When the lighting conditions are known, the reflected light distributions from an object can be calculated with its BRDF (Bidirectional Reflection Distribution Function), for which several models exist. In photographic applications (Supplementary Fig. 1c), an object renders approximately the same image intensity across different propagation angles (hence sub-apertures) because the angular range covered by the lens system is typically small. For example, given an image magnification of 0.01 and *f-number* of 2.0 (a fast lens in photography), the NA at the object side is 0.0025, spanning an angle of only 0.045 degrees. Except for mirror-like specular objects that show highly directional BRDF, the recorded light intensity variations along different angles are negligible compared to that induced by the lens' vignetting effects. In sum, ignoring the edge pixels that may lose correspondence among sub-aperture images, the image $p(x, y, u_k, v_k)$ observed from view (u_k, v_k) can be related to a reference sub-aperture image $p(x, y)$ by:

$$p(x, y, u_k, v_k) = p(x - su, y - sv), \quad (3)$$

which can be represented by an invertible matrix $\mathbf{B_k}$ as $\mathbf{h_k} = \mathbf{B_k h}$, with $\mathbf{h_k}$ and $\mathbf{h}$ denoting the vectorized sub-aperture image $p(x, y, u_k, v_k)$ and reference image $p(x, y)$, respectively.

The implicit assumption of uniform angular intensity can also be valid in microscopic imaging. For instance, the fluorophores used in fluorescence microscopy emit light in an omnidirectional manner, with a uniform intensity distribution across all directions. For scenarios where this assumption becomes invalid, CLIP can still correctly recover the scene geometry but with inaccurate intensities, preventing quantitative image analysis. We show in Supplementary Note 3 that such implicit assumptions are common in computational cameras that attain a subset of light field imaging capabilities.

Supplementary Note 2. CLIP working flow

We summarize CLIP's working flow of light field processing in Supplementary Fig. 2. The core capability of a light field camera is post-capture refocusing—other functionalities such as extending depth of field (eDOF) and depth retrieval are built upon it, and their associated processing algorithms are well documented in the literature.

a) Refocusing is done by regenerating the system model **F(d)** for a specified shearing factor s and then performing image reconstruction.

b) Viewpoint synthesis is achieved by changing the reference sub-aperture (N_{refx}, N_{refy}) during generation of the system model **F(d)**. The reference sub-aperture (N_{refx}, N_{refy}) can be a virtual one and not necessarily of an integer number, allowing CLIP to synthesize novel viewpoints not present during data acquisition.

c) Computationally extending the depth of field is similar to conventional light field cameras: by refocusing onto different depths and extracting for each pixel the sharpest feature, CLIP can assemble an all-in-focus image[1] to extend the depth of field.
d) Depth retrieval. While the nonlocal acquisition of implicit light field data in CLIP prevents disparity information from being directly extracted from the measurement data for depth extraction (such as forming an epipolar image), the same end can be achieved by the depth from focus (DfF) method[2]. As in conventional approaches, this requires the images to show enough texture/features to make depth retrieval feasible.

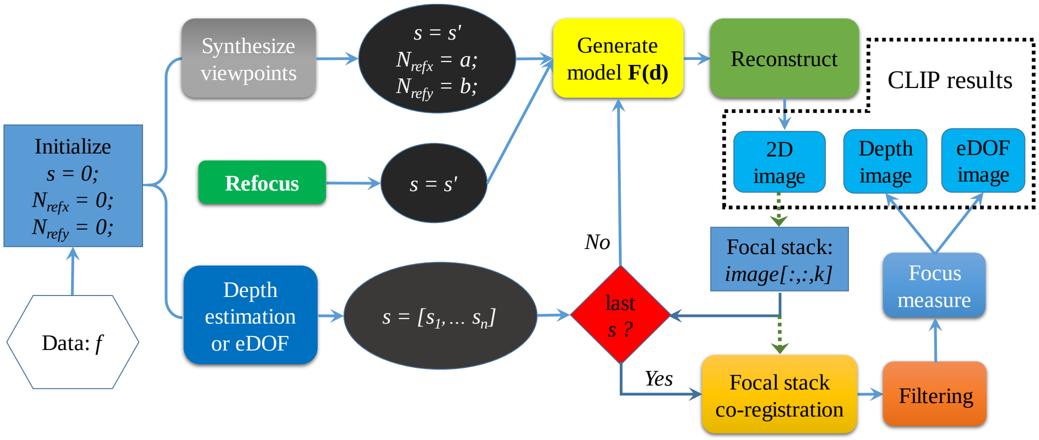

Supplementary Figure 2. **Working flow of CLIP.** The core is to regenerate the system's forward model **F(d)** when the refocusing parameter *s* and/or reference sub-aperture (N_{refx}, N_{refy}) are changed (for novel view synthesis). Afterward, the image is reconstructed. Depth estimation and extending depth of field (eDOF) need to refocus on *n* different depths (for a depth resolution of *n*) to generate a focal image stack. The depth yielding the maximum focus measure (a measure of image contrast) is identified across the focal stack on a pixel basis. Assembling the sharpest feature into a single image leads to an extended DOF while assigning each pixel with its detected depth renders a relative depth image.

Supplementary Note 3. CLIP with 2D area sensors

Light field imaging with 2D sensors without compromising image resolution is well studied, with a wealth of techniques including coded aperture[3] and wavefront coding[4] exist. Recovering a full 4D light field (*Na*, *Na*, *N*, *N*) from a densely sampled 2D image (*N*, *N*) has also been achieved by the compressive[5] and diffuser-encoded light field camera[6,7]. Here, we show that dealing with a sparse 2D sensor becomes more demanding that makes most existing designs inadequate. Additionally, the coded-aperture, wavefront-coding, and diffuser-based methods can be unified into the CLIP framework, as the assumption of uniform angular intensity proves to be the sufficient condition for their validity.

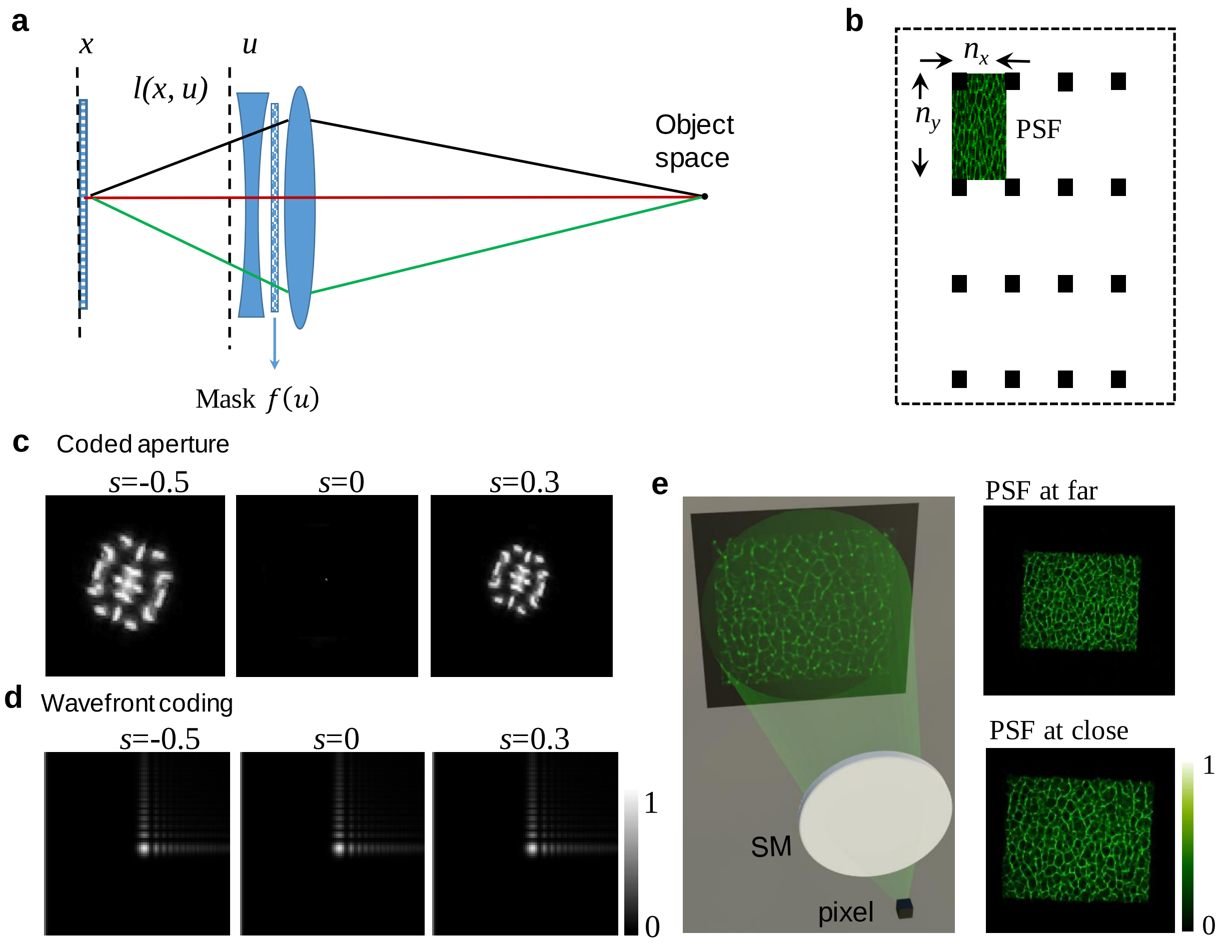

Supplementary Figure 3. CLIP with 2D area detectors. a Computational cameras with coded aperture and wavefront coding. The light field is parameterized with the spatial axis *x* on the sensor plane and the angular *u* axis on the aperture. A complex-valued mask is inserted in the aperture plane to modulate the light field. **b** When the 2D sensor is sparsely packed, the PSF needs to be larger than the pixel pitch such that all the scene information is encoded into the measurement, which is a down-sampled convolution of the scene and system PSF. **c** The PSFs at different depths for the coded-aperture camera. Note that for any amplitude code in the aperture, the PSF is a Dirac function at *s=0*. **d** The PSF at different depths for wavefront-coding with a cubic phase plate. The PSFs are approximately depth-invariant as the cubic phase plate is designed to extend the depth of field. **e** Diffuser camera uses a thin scattering medium for lensless imaging. The PSF is a random caustic pattern that remains shift-invariant on an image plane within an angular range determined by the memory effect. The PSF is scaled at different imaging depths. SM: scattering medium. PSF: point spread function; *s*: refocusing parameter.

In coded aperture and wavefront coding methods, an amplitude or phase mask is placed at the aperture to modulate the light field, as shown in Supplementary Fig. 3a. More generally, the mask can be a complex-valued function to modulate both the amplitude and phase of the transmitted light, as does by a scattering medium. Without loss of generality, we confine the light field analysis to 2D (one spatial axis and one angular axis) and assume the system to be shift-invariant such that the imaging process is convolutional with a system kernel $r(x, u)$ in the ray space. Denoting the

light field as $p(x,u)$, which is essentially a sub-aperture image observed from an infinitesimal patch around u on the aperture, the sensor measurement can be written as[8]:

$$\begin{aligned} i(x) &= r(x,u) *_{x,u} p(x,u) \\ &= \int r(x, 0-u) *_x p(x,u) du \\ &= \int r(x,-u) *_x p(x-su) du \\ &= \int r(x,-u) *_x p(x) *_x \delta(x-su) du \\ &= p(x) *_x \int r(x,-u) *_x \delta(x-su) du \\ &= p(x) *_x \int r(x-su,-u) du \\ &= p(x) *_x g(x,s)\,, \end{aligned} \qquad (4)$$

where $*_x$ and $*_{x,u}$ denote convolution along the *x*-axis and in the 2D ray space, respectively. $g(x,s) = \int r(x-su,-u)du$ is the system's PSF at the depth indexed by s. Note that the uniform angular intensity assumption $p(x,u) = p(x-su) = p(x) *_x \delta(x-su)$ has been applied during the derivation. The sensor measurement is, therefore, the convolution of the reference image with a depth-dependent PSF. The model reduces to CLIP in the discrete domain:

$$\begin{aligned} i(x) &= \int_{u_0}^{u_N} r(x,-u) *_x p(x) *_x \delta(x-su) du \\ &= \sum_{i=0}^{N-1} \int_{u_k-\Delta u/2}^{u_k+\Delta u/2} r(x,-u) *_x p(x) *_x \delta(x-su) du \xrightarrow{mean\ value\ theorem} \\ &= \Delta u \sum_{k=0}^{N-1} r(x,-u_k) *_x p(x) *_x \delta(x-su_k) \\ &= \sum_{k=0}^{N-1} \underbrace{r'(x,-u_k) *_x}_{\mathbf{A_k}} \underbrace{[\delta(x-su_k) *_x p(x)]}_{\mathbf{B_k}h} \xrightarrow{Discretize\ convolution} \\ &= \textstyle\sum_{k=0}^{N-1} \mathbf{A_k B_k h} = \underbrace{\mathbf{T}\begin{bmatrix} \mathbf{A_1B_1} \\ \mathbf{A_2B_2} \\ \vdots \\ \mathbf{A}_N\mathbf{B}_N \end{bmatrix}}_{\boldsymbol{Convolution\ matrix}} \mathbf{h} = \mathbf{F(d)}\, h = \mathbf{f} \end{aligned} \qquad (5)$$

where $r'(x,-u_k) = r(x,-u_k)\Delta u$, and $\mathbf{T} = [\mathbf{I}, \mathbf{I}, \cdots\ \mathbf{I}]$ is the integration operator that preserves the Toeplitz structure of matrix $\mathbf{A_k B_k}$ and multiplex the sub-aperture measurements $\mathbf{A_k B_k h}$ into a single image measurement. Notably, the convolutional model of Supplementary Eq. (4-5) applies indiscriminately to computational cameras using a diffuser, coded aperture, or wavefront coding, and the image recovery is essentially a deconvolution problem. We illustrate the agreement with existing results[3,9,10] by examining a coded aperture camera with an amplitude mask $f(u)$. The mask together with the lens system yields a kernel $k(x,u) = f(u)\delta(x)$ in the ray space, leading to a depth-dependent PSF that is calculated as:

$$g(x,s) = \int k(x - su, -u)du = \int f(-u)\delta(x - su)du = sf\left(-\frac{x}{s}\right). \quad (6)$$

Substituting into Supplementary Eq. (4), the sensor measurement is:

$$i(x) = sf\left(-\frac{x}{s}\right) *_x p(x), \quad (7)$$

which is the same model of coded aperture cameras[3]—the recorded photograph is the convolution of a scaled aperture function $f\left(-\frac{x}{s}\right)$ with an ideal image. For instance, focusing at nominal focal plane is obtained by setting $s=0$, with which the aperture function is scaled to a Dirac delta function. Refocusing onto another depth involves re-scaling the aperture function and applying a deconvolution step to recover the image, equivalent to regenerating the depth-dependent model **F(d)** in CLIP and subsequently reconstructing a refocused image.

Under the convolutional model, few of these existing techniques can be directly applied to sparsely packed 2D detectors or sensors of lower dimensionality (0D and 1D). With a sparse detector that has a pixel pitch of n_x and n_y (in the unit of pixel size), the measurement is a down-sampled version of the convolution results $i(x)$ with a factor of n_x and n_y along the two spatial axes. By reciprocity, the system PSF $g(x,s)$ is the measurement pattern of an individual pixel for the scene at the depth indexed by s. To encode the complete 3D scene into the sensor measurement, the pixel pitch must be smaller than the size of PSFs at all depths, as illustrated in Supplementary Fig. 3b. Moreover, to ensure the image reconstruction to be well-posed, the system PSFs at different depths need to show non-negligible high-frequency features[3].

With this in mind, the coded-aperture methods hence demand a properly sampled 2D photograph because its PSF is a Dirac function at $s=0$ (Supplementary Fig. 3c). For wavefront coding methods, current designs typically yield relatively sparse PSFs (Supplementary Fig. 3d for the cubic phase plate that yields depth-invariant PSFs) with a compact size (compared to the image size) at all depths, allowing a small downsampling factor (or sparsity) for 2D sensors but remain infeasible to cover the entire scene with a 1D sensor. By contrast, the random PSF produced by a scattering medium (a caustic pattern for a thin diffuser[6,7], Supplementary Fig. 3d) densely covers a large image area with high-frequency features at all depths. This makes it suitable for light field imaging using a sparsely packed 2D sensor. Nevertheless, we show in the next section that while theoretically feasible, the diffuser camera approach to perform 2D imaging with a 1D sensor[11] is ill-posed and far from optimal.

We demonstrate the feasibility of CLIP imaging using 2D sensors (dense and sparse) via synthetic studies in Supplementary Figure 4, using a diffuser at the aperture (like DiffuserCam[9]) as an example implementation. The imaging performance for scenes of different complexities is investigated at a fixed depth. The full measurement data is generated by convolving a ground-truth image with the system PSF. Measurement using sensors with different sparsity (sparsity of 1 is equivalent to a dense sensor) is simulated by downsampling the full measurement correspondingly and adding 2.5% white noise to emulate sensor noises.

Supplementary Figure 4a-c shows the imaging results for the complex "monkey" image, a resolution target, and a simple letter image, respectively. The sensor sparsity is varied from 1 (Nyquist sampling) to 16 across both spatial axis from left to right, leading to a measurement data size ranging from 100% to only 0.4% of the full image size. The random wide-field PSF permits a sparsity factor of 2 for the recording sensor to attain a good image recovery for the complex monkey image. As the sparsity becomes more significant, more high-frequency image details get

washed out, but the low-frequency structure of the image is still obtained. For simpler (more compressible) images, however, a greater sparsity factor can be accommodated. For the letter image, in particular, a measurement with a sparsity factor of 8 (~1.6% of complete image data) still managed to recover it despite of some burring. The ability to handle sparse detectors also proves the robustness of CLIP against defective sensor readings, which typically cause only a small faction of the measurement data to be lost.

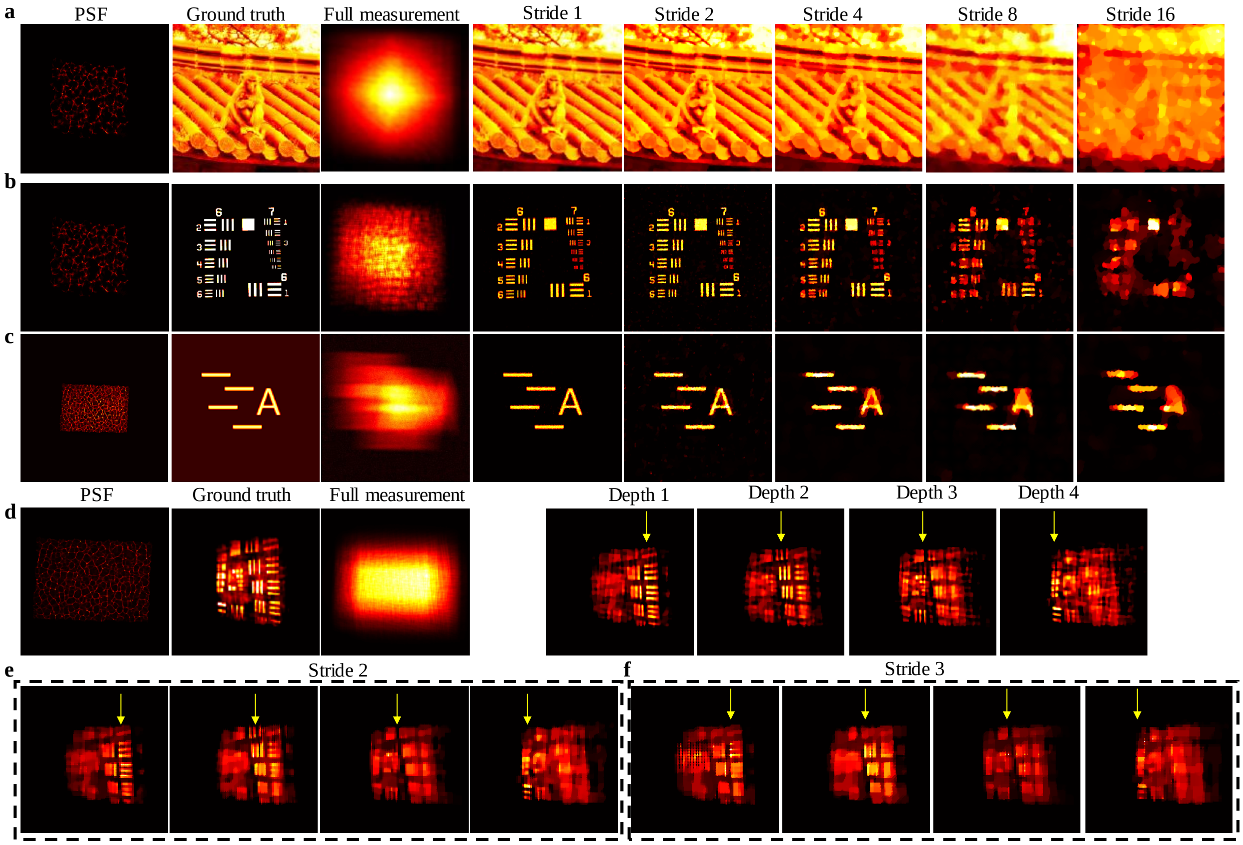

Supplementary Figure 4. CLIP imaging with 2D sensors. a-c CLIP imaging for the monkey image, the resolution target, and the simple letter image with different sampling sparsities. From left to right are the random wide-field PSFs, the ground truth image, the full measurement data, and the reconstruction results with a sparsity factor of 1, 2, 4, 8, and 16 for the 2D sensor. **d** Reconstruction of the slanted resolution target in the pubic DiffuserCam dataset by CLIP at different refocusing depths. **e-f** The same reconstruction of the slanted resolution target after downsampling the measurement by 2 and 3 times. PSF: point spread function.

We further validated CLIP on the publicly released DiffuserCam[9] dataset, which share the same setup as the synthetic study in employing a diffuser for 3D imaging. It originally aimed to directly retrieve a 3D volumetric scene from a dense 2D measurement. For CLIP, we recover only a refocused image by solving Supplementary Eq. (5) with a PSF at the corresponding depth. Supplementary Figure 4d shows the CLIP reconstructed images refocused at different depths for the slanted resolution target, along with the projected 2D image from the original algorithm for comparison. To demonstrate the capability to cope with a sparse 2D detector, we down-sampled

the experimental measurement by 2 and 3 times and then reconstructed the image at the same refocusing depths in Fig. 4e-f, respectively. It is noted that CLIP can reconstruct the resolution target with a sparsity factor of 2 with the refocusing effect being observed in all the cases, validating the capability of CLIP to handle 2D sparse detectors. However, the images get blurred for the sparsity factor of 3, similar to the observation made in the simulation results.

Supplementary Note 4. CLIP imaging with 1D sensors

With the convolutional imaging model, we show that performing 2D imaging with a 1D sensor is an ill-posed problem. According to the compressive sensing theory[12], the number of measurements required for recovering a s-sparse signal (i.e., having s non-zero coefficients in a suitable representation basis) of size N is $M = O(slogN)$, provided that an appropriate measurement scheme is employed. While a 1D sensor with N pixels seems feasible to recover a 2D image of $N \times N$ with a sparsity of $s = O(N/(2logN))$, the sampling scheme as performed by the 1D sensor proves to be inadequate to approach the bound. Following Eq. (4), the 1D sensor measurement is a single slice of the convolutional results:

$$i(x, y = 0) = p(x, y) *_x g(x, y, s)|_{y=0}. \quad (8)$$

In the Fourier domain, it can be written as:

$$L(k_x) = \int P(k_x, k_y) G(k_x, k_y, s)\, dk_y = \int I(k_x, k_y)\, dk_y, \quad (9)$$

which states that the Fourier transform of the 1D sensor measurement $L(k_x)$ is equivalent to a projection of the image spectrum $I(k_x, k_y)$ along the k_y axis in the Fourier domain, a dual to the Fourier slice theorem. We prove this equation below. Denoting the Fourier transform operation as FT and the inverse transform as FT^{-1}, we can obtain (omitting the 2π constant throughout):

$$\begin{aligned} FT^{-1}(L(k_x)) &= \int L(k_x) e^{i2\pi k_x x}\, dk_x \\ &= \int \int I(k_x, k_y)\, dk_y\, e^{i2\pi k_x x} dk_x \\ &= \int \int I(k_x, k_y)\, e^{i2\pi k_y \times 0} dk_y\, e^{i2\pi k_x x} dk_x \\ &= \int \int I(k_x, k_y)\, e^{i2\pi k_y \times 0}\, e^{i2\pi k_x x} dk_x dk_y \\ &= i(x, y = 0)\,. \quad (10) \end{aligned}$$

Selecting a different 1D slice of the image *i(x, y)* can be done by multiplying the image spectrum $I(k_x, k_y)$ with a proper phase function in Supplementary Eq. (10) as:

$$\begin{aligned} FT^{-1}(L(k_x)) &= \int \int I(k_x, k_y)\, e^{i2\pi k_y \times n}\, e^{i2\pi k_x x} dk_x dk_y \\ &= i(x, y = n)\,. \quad (11) \end{aligned}$$

Following the same analysis, we can further prove that extracting a single-pixel measurement from an image is equivalent to a 2D summation or projection of the spectrum:

$$\iint I(k_x, k_y)\, dk_x dk_y = \int \iiint i(x,y) e^{-i2\pi k_x x} e^{-i2\pi k_y y} dx dy\, dk_x dk_y$$
$$= \iint \iint e^{-i2\pi k_x x} dk_x e^{-i2\pi k_y y}\, dk_y i(x,y) dx dy$$
$$= \iint \delta(x)\delta(y)\, i(x,y) dx dy$$
$$= i(x = 0, y = 0)\,. \quad (12)$$

Supplementary Eq. (9) can be written in the matrix formalism as:

$$\mathbf{L} = \int G(k_x, k_y, s) P(k_x, k_y)\, dk_y = \mathbf{GP}, \qquad (13)$$

with **P** being the vectorized image spectrum. **G** is a matrix whose row vector is the column entry of $G(k_x, k_y, s)$:

$$\mathbf{G} = \begin{bmatrix} \mathbf{G_1^T} & \mathbf{0} & \mathbf{0} & \mathbf{0} \\ \mathbf{0} & \mathbf{G_2^T} & \mathbf{0} & \mathbf{0} \\ \mathbf{0} & \mathbf{0} & \mathbf{G_3^T} & \mathbf{0} \\ \vdots & \vdots & \vdots & \vdots \\ \mathbf{0} & \mathbf{0} & \mathbf{0} & \mathbf{G_n^T} \end{bmatrix}, \qquad (14)$$

where $\mathbf{G_j}$ is the j-th column of $\mathbf{G}(k_x, k_y, s)$. Hence, each column of the image spectrum is independently coded by G_j and then integrated:

$$\mathbf{L}[j] = \mathbf{G_j^T P_j}. \qquad (15)$$

This reveals the ill-posed nature of the problem of 2D imaging with 1D sensors: it essentially involves recovering a column $\mathbf{P_j}$ from a single measurement $\mathbf{L}[j]$. Even with a strong prior that the signal $\mathbf{P_k}$ is one-sparse, the minimum number of measurements needed is $M = O(logN)$, indicating the inversion of Supplementary Eq. 15 will be unreliable.

CLIP's approach to improving the conditioning of imaging with 1D sensors is to use a different PSF $G(k_x, k_y, s)$ for each sub-aperture measurement, and thus increase the incoherent measurement number M:

$$\begin{bmatrix} \mathbf{L_1}[\mathrm{j}] \\ \vdots \\ \mathbf{L_M}[\mathrm{j}] \end{bmatrix} = \begin{bmatrix} \mathbf{G_{1_j}}^{\mathbf{T}} \\ \vdots \\ \mathbf{G_{M_j}}^{\mathbf{T}} \end{bmatrix} \mathbf{P_j}. \qquad (16)$$

To design PSFs that are close-to-optimal for imaging with 1D sensors, we first identify the desired imaging metrics to be optimized: 1) a small PSF size N_p in the spatial domain, and 2) incoherent PSFs with broad bandwidth. The first is to maximize image resolution for a given sensor resolution N_x—the maximum image resolution that the sensor can accommodate for a PSF of size N_p is N=N_x-N_p. The incoherent PSFs with broad bandwidth are to satisfy the multiplexing requirement for stable signal recovery. As exemplified in Supplementary Eq. 16, the PSF column ${G_1}_j^T$ need be both broadband to efficiently encode P_j into the measurement and be mutually incoherent to ensure a well-conditioned matrix for a stable solution. The minimal N_p is 1, which corresponds to line-shaped PSFs in both the spatial and Fourier domains, as indicated in Supplementary Fig. 5a. To make them mutually incoherent and broadband, the line-shaped PSFs are rotated to different angles

and further modulated by random codes to spread the spectrum over the entire Fourier domain (Supplementary Fig. 5b). For reference and comparison, randomly coded PSFs of various sizes N_p (2, 4, 8, and 128) and their corresponding spectra are shown in Supplementary Figure 5c. Random coding of the line-shaped PSF can be implemented by attaching a coded amplitude mask onto a cylindrical lens as in Supplementary Fig. 5d. Because random codes are the optimal sensing basis when no prior signal information is available[12], randomly coded line-shaped PSF is close to optimal for imaging with 1D sensors.

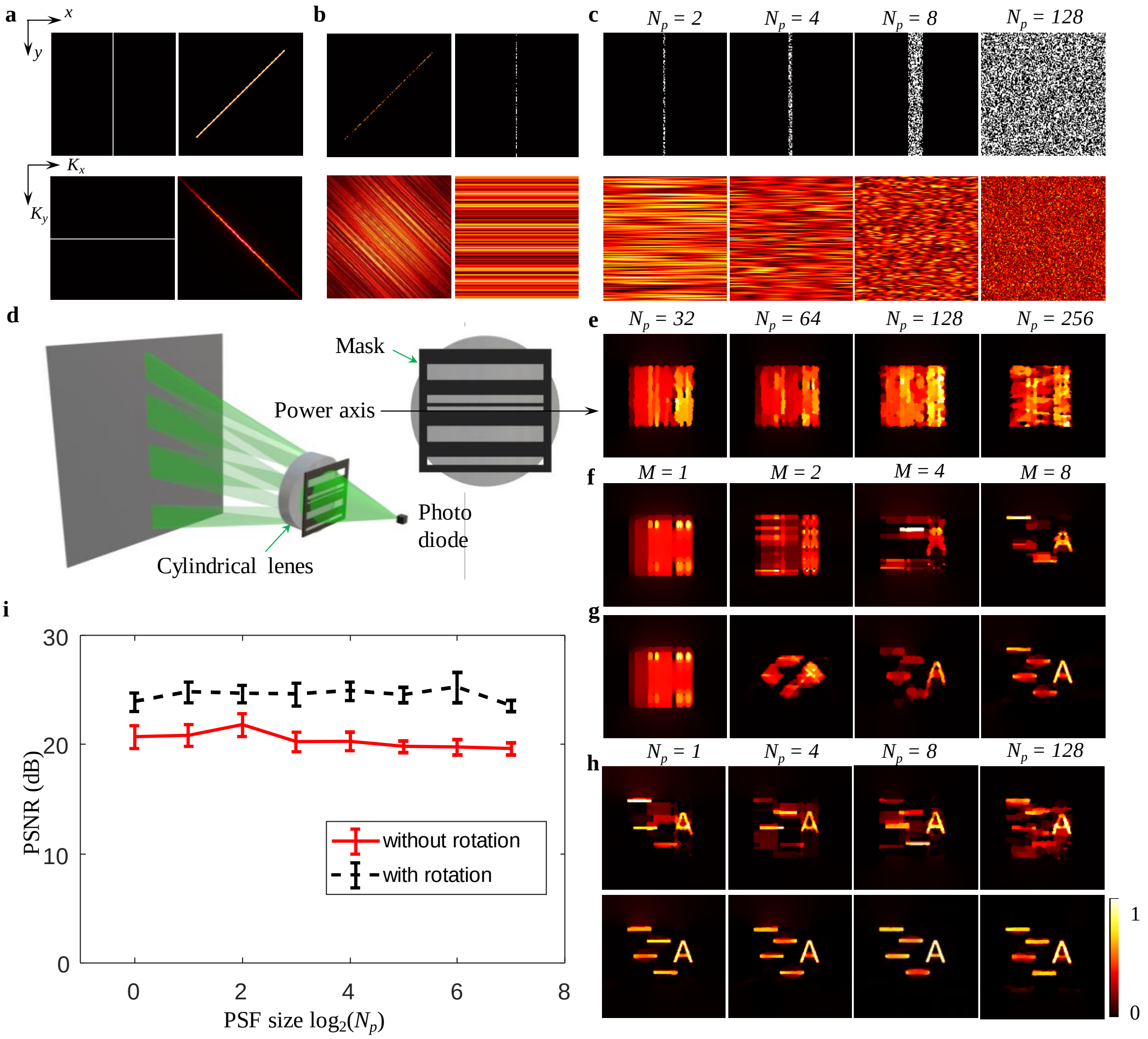

Supplementary Figure 5. CLIP imaging with 1D sensors. a The line-shaped PSFs (top row) at different rotation angles and their corresponding spectrum in the Fourier domain (bottom row). **b** Encrypting the line-shaped PSF with random binary codes spreads the spectrum to cover the entire Fourier domain to obtain efficient multiplexing of the measurement for compressive sensing. **c** PSFs with different spatial sizes and encrypted with random codes to promote broadband multiplexing. The corresponding spectra are shown in the bottom rows. **d** The encrypted line-shaped PSF can be implemented by coding the cylindrical lens aperture with an (essentially one-dimensional) amplitude

mask. **e** Image reconstruction with a 1D sensor and a PSF with various spatial sizes N_p (1 to 256). **f** Fix the PSF size at N_p =256 and reconstruct the image with different numbers of measurement M (1 to 8). **g** Image reconstruction by rotating the corresponding PSFs in **f** to different angles in the range of [-45°, 45°]. **h** Reconstructed images using M=10 different PSFs of different sizes without (top) and with (bottom) rotations. **i** The reconstruction quality is measured by the *PSNR*. The vertical lines at each point is the error bar. Note that the random line-shaped PSF with rotations consistently obtains the best reconstruction quality. *PSNR*: peak signal to noise ratio; PSF: point spread function; N_p: line width.

We validated the optimality of randomly codeds line-shaped PSFs via synthetic studies. Imaging with a single random PSF with various sizes N_p for the simple letter scene is given in Supplementary Fig. 5e. As expected, the ill-posed nature of the problem prevented decent image recovery in this case. However, when one gradually increases the measurement number M (i.e., M PSFs) as in Supplementary Fig. 5f, the images are recovered with progressively better fidelity. Nevertheless, noticeable artefacts are still observed in the reconstructions. By rotating the PSFs uniformly into different angles in the range of [-45°, 45°], the image quality in Supplementary Fig. 5g is drastically improved. We evaluated the reconstructed image quality by the peak signal-to-noise ratio (PSNR) and quantified the improvement obtained by CLIP in Supplementary Fig. 5h-i. For this particular quantitative study, the number of PSFs is fixed at M=10, and we also varied the PSFs size N_p to show the close-to-optimality of line-shaped PSFs. The image reconstructions without and with applying the PSF rotation are given in the top and bottom rows of Supplementary Fig. 5h. And the PSNR of the reconstructions is depicted in Supplementary Fig. 5i. It is noted that the PSF size doesn't affect the reconstruction quality, while applying rotations to the PSF consistently improves the quality by more than 4 dB in PSNR. Overall, randomly coded line-shape PSF attains the best reconstruction quality while allowing the most efficient utilization of the sensor pixels, proving its close-to-optimal performance for imaging with 1D sensors.

It is noted that the implementation for randomly coded line-shape PSF is very similar to the coded-aerpture camera, with the camera lens and image sensor being replaced by a cylindrical one and 1D sensor respectively. Like coded-aperture imaging therefore, a one-time calibration step for the camera will be needed to retrieve PSF on the sensor by imaging a point source and scanning the 1D sensor along the other dimension.

Supplementary Note 5. Comparison of CLIP with compressive light field photography

Existing compressive light field imaging methods are not necessarily convolutional and can recover a 4D light field ($n_a \times n_a \times N \times N$) from a 2D image ($N \times N$). We compare them with CLIP and explain the unique advantages of CLIP in using sensors of arbitrary formats for efficient light field imaging. Most compressive light field photography methods share the roots with coded aperture imaging in using a mask (transmissive or reflective) to divide the system aperture into small patches, each modulating a sub-aperture image. The resultant sensor measurement is a weighted integration of all the sub-aperture images:

$$\mathbf{y_1} = \sum_{k=1}^{n_a^2} w_{1k}\mathbf{P_k} = \left[w_{11}\mathbf{I},\ w_{12}\mathbf{I}, \cdots, w_{1n_a^2}\mathbf{I}\right] \begin{bmatrix} \mathbf{P_1} \\ \mathbf{P_2} \\ \vdots \\ \mathbf{P}_{n_a^2} \end{bmatrix} \qquad (17)$$

where $\mathbf{y_1} \in \mathbb{R}^{n^2\times 1}$ is the vectorized sensor image, $\mathbf{I} \in \mathbb{R}^{n^2\times n^2}$ is the identity matrix. $w_{1k} \neq w_{1j}$, and it is a scalar representing the mask transmission coefficient for the *k-th* sub-aperture image. $\mathbf{P}_{\boldsymbol{k}} \in \mathbb{R}^{\boldsymbol{n}^2\times \mathbf{1}}$ is the corresponding vectorized sub-aperture image. It is noted that imaging without the coding mask is equivalent to setting all the weights w_{1k} to 1. While n_a^2 different set of mask coefficients w_{jk} (and sensor measurements y_j) are typically needed to recover the light field ($\mathbf{P_1}$ to $\mathbf{P}_{\boldsymbol{n_a^2}}$), Ashok[13] and Babacan[14] proposed to use a smaller number $m<n_a^2$ of mask coefficients and relied on the sparsity prior for a compressive reconstruction of a 4D light field. Ashok et.al., further showed that one can use a similar coding scheme for each microlens in an unfocused light field camera, and recover the spatial image on the microlens with a sub-Nyquist measurement dataset, thereby addressing the angular-spatial resolution tradeoff in unfocused light field cameras. Nevertheless, multiple measurements are still needed in Ashok and Babacan's methods for recovering a light field.

Marwah[5] et.al., generalized the mask position to anywhere between the aperture and the sensor. When the mask is positioned close to the sensor, different sub-aperture images are modulated with sheared (and thus incoherent) mask codes before being integrated by the sensor:

$$\mathbf{y} = \sum_{k=1}^{n_a^2} \mathbf{C_k P_k} = \left[\mathbf{C_1}, \mathbf{C_2}, \cdots, \mathbf{C}_{n_a^2}\right] \begin{bmatrix} \mathbf{P_1} \\ \mathbf{P_2} \\ \vdots \\ \mathbf{P}_{\boldsymbol{n_a^2}} \end{bmatrix} \quad (18)$$

where $\mathbf{C_k} \in \mathbb{R}^{n^2\times n^2}$ is the block diagonal matrix containing the sheared mask code. One key improvement of Marvah's work lies in the modulation of each sub-aperture image $\mathbf{P}_{\boldsymbol{k}}$ with a random code $\mathbf{C_k}$ rather than $w_{jk}\mathbf{I}$ as in Supplementary Eq. 17, thereby improving the conditioning of the inverse problem as $\mathbf{C_k}$ is incoherent with respect to each other. Coupled with a dictionary learning process that better sparsifies a 4D light field, Marwah's approach can recover a full 4D light field from a single measurement, eliminating the need of changing the mask codes.

The diffuser-camera-based light field imaging[6,7] differs from the above approaches in being convolutional: each sub-aperture image is convolved with a random nonlocal point-spread-function (PSF) before integration:

$$\mathbf{y} = \sum_{k=1}^{n} \mathbf{M_k P_k} = \left[\mathbf{M_1}, \mathbf{M_2}, \cdots, \mathbf{M}_{n_a^2}\right] \begin{bmatrix} \mathbf{P_1} \\ \mathbf{P_2} \\ \vdots \\ \mathbf{P}_{\boldsymbol{n_a^2}} \end{bmatrix} \quad (19)$$

with $\mathbf{M_k} \in \mathbb{R}^{n^2\times n^2}$ being the Toeplitz convolution matrix for the random PSF in the *k*-th angular view. Light field imaging based on diffuser camera can be implemented with both lens[8] and lensless manners[7]. When being used with a lens, the PSF for each sub-aperture image is more compactly supported, leading to an efficient utilization of the sensor pixels owing to smaller boarder effects. In contrast, the lensless approach features system simplicity, and it is free from lens-aberrations.

It is now clear that the differentiating factor among existing compressive light field imaging methods is the matrix operating on each sub-aperture image. The matrices ($\mathbf{I}$, $\mathbf{C_k}$) in Ashok, Babacan, and Marwah et.al. are all diagonal. As a result, the sensor resolution directly determines the spatial resolution of the recovered light field (both $\mathbf{y}$ and $\mathbf{P}_{\boldsymbol{k}}$ are in $\mathbb{R}^{n^2\times 1}$), making these methods ill-suited for 0D, 1D, and sparse 2D sensors. In contrast, the Toeplitz matrix $\mathbf{M_k}$ in

diffuser-camera-based light field imaging is non-diagonal, and its row vectors multiplex multiple elements of $\mathbf{P}_k$ into one measurement in $\mathbf{y}$ (owing to a nonlocal PSF). Though not being demonstrated yet, this allows in theory the recovery of a 4D light field from a sub-Nyquist measurement dataset (that is $\mathbf{y} \in \mathbb{R}^{m\times 1}$ with $m < n^2$while $\mathbf{P_k} \in \mathbb{R}^{n^2\times 1}$).

In contrast, CLIP is a systematic method for designing and transforming any imaging methods with nonlocal data acquisition into a highly efficient light field imaging approach. For a given imaging model with measurement matrix $\mathbf{A}$, the transformation of CLIP is achieved by splitting the measurements into different angular views, as illustrated below:

$$\mathbf{y} = \mathbf{Ax} = \begin{bmatrix} \mathbf{a_1^T} \\ \mathbf{a_2^T} \\ \vdots \\ \vdots \\ \mathbf{a_l^T} \end{bmatrix} \mathbf{x} \;\underbrace{\xRightarrow[\qquad]{\textbf{Transforming}: \textit{measurement splitting}}}_{\textit{CLIP Step } 1}$$

$$\mathbf{y} = \begin{bmatrix} view\_1\left\{\begin{bmatrix} \mathbf{a_1^T} \\ \vdots \\ \mathbf{a_q^T} \end{bmatrix}\right. & \cdots & \mathbf{0} \\ \vdots & view\_k\left\{\begin{bmatrix} \mathbf{a_{kq+1}^T} \\ \vdots \\ \mathbf{a_{kq+q}^T} \end{bmatrix}\right. & \vdots \\ \mathbf{0} & \ddots & \mathbf{0} \\ \mathbf{0} & \cdots & view\_l\left\{\begin{bmatrix} \mathbf{a_{lq+1}^T} \\ \vdots \\ \mathbf{a_{lq+q}^T} \end{bmatrix}\right. \end{bmatrix} \begin{bmatrix} \mathbf{P_1} \\ \mathbf{P_2} \\ \vdots \\ \mathbf{P}_l \end{bmatrix} = \begin{bmatrix} \mathbf{A_1} & \cdots & \mathbf{0} \\ \vdots & \mathbf{A_2} & \vdots \\ \mathbf{0} & \ddots & \mathbf{0} \\ \mathbf{0} & \cdots & \mathbf{A}_l \end{bmatrix} \begin{bmatrix} \mathbf{P_1} \\ \mathbf{P_2} \\ \vdots \\ \mathbf{P}_l \end{bmatrix} = \mathbf{A}' \begin{bmatrix} \mathbf{P_1} \\ \mathbf{P_2} \\ \vdots \\ \mathbf{P}_l \end{bmatrix}, \quad (20)$$

where $\mathbf{a_k^T}$ is a row vector and $\mathbf{x}$ (an image from a single angular view) is extended to a 4D light field ($\mathbf{P_1}$ to $\mathbf{P}_l$) with l=n_a^2 views (sub-apertures). While the imaging model becomes bock diagonal, recovering the light field is equivalent to solve each sub-aperture image $\mathbf{P_k}$ with a corresponding sub-measurement matrix $\mathbf{A_k}$. We can better exploit the correlations (redundancy) in the 4D light field by solving Supplementary Eq. 20 with appropriate sparsity based regularizations, as used in compressive light field imaging methods[5–7]. It is noteworthy that the elemental matrix $\mathbf{A_k}$ is not longer diagonal as $\mathbf{I}$ or $\mathbf{C_k}$, a key fact that enables CLIP to use 0D or 1D sensors for light field imaging. We demonstrated 4D light field recovery using CLIP in Supplementary Note 7.

The second key differentiating factor of CLIP is explicit modeling of the correlations among sub-aperture images as $\mathbf{P}_k = \mathbf{B_k h}$ via light field propagation, assuming a uniform angular intensity distribution as derived in Supplementary Note 1. This simplifies Supplementary Eq. 20 to the CLIP equation 3 in the main text:

$$\mathbf{y} = \begin{bmatrix} \mathbf{A_1} & \cdots & \mathbf{0} \\ \vdots & \mathbf{A_2} & \vdots \\ \mathbf{0} & \ddots & \mathbf{0} \\ \mathbf{0} & \cdots & \mathbf{A}_l \end{bmatrix} \begin{bmatrix} \mathbf{P_1} \\ \mathbf{P_2} \\ \vdots \\ \mathbf{P}_l \end{bmatrix} \underbrace{\xRightarrow{\mathbf{P_k}=\mathbf{B_k h}}}_{\textit{CLIP Step}2} \mathbf{y} = \begin{bmatrix} \mathbf{A_1} & \cdots & \mathbf{0} \\ \vdots & \mathbf{A_2} & \vdots \\ \mathbf{0} & \ddots & \mathbf{0} \\ \mathbf{0} & \cdots & \mathbf{A}_l \end{bmatrix} \begin{bmatrix} \mathbf{B_1 h} \\ \mathbf{B_2 h} \\ \vdots \\ \mathbf{B}_l\mathbf{h} \end{bmatrix} = \begin{bmatrix} \mathbf{A_1 B_1} \\ \mathbf{A_2 B_2} \\ \vdots \\ \mathbf{A}_l\mathbf{B}_l \end{bmatrix} \mathbf{h} = \mathbf{A}''\mathbf{h}. \quad (21)$$

This step has the advantage of enabling more complicated images to be recovered without the need of finding/learning a better sparsifying basis for the 4D light field, which is an important step in Marwah's work. We show this advantage in Supplementary Note 8.

The computation complexity of compressive light field photography and CLIP depends on the light field resolution and the applied regularization method under the framework of regularization by denoising (see **Methods)**. In CLIP, each iteration involves a pass of $\mathbf{A}''$ and $\mathbf{A}''^{\boldsymbol{T}}$ along with a denoising step. The complexity for the shearing operation and matrix $\mathbf{A}$ is $\mathrm{o}(n_a^2 N^2)$ and $\mathrm{o}(mN^2)$ respectively, leading to a total complexity of $\mathrm{o}\big((n_a^2 + m)N^2\big)$ for both $\mathbf{A}''$ and $\mathbf{A}''^{\boldsymbol{T}}$. The complexity of BM3D and TV denoising for regularization is directly related to the image size as $\mathrm{o}(kN^2)$, with k being a denoiser-dependent constant. Therefore, the total complexity of CLIP image recovery is $\mathrm{o}\big((2m + 2n_a^2 + k)N^2\big)$ per iteration. In comparison, while the complexity for $\mathbf{A}'$ and $\mathbf{A}'^{\boldsymbol{T}}$ in Supplementary Eq. 20 for retrieving the 4D light field remains $\mathrm{o}(mN^2)$ owing to the block diagonal structure, the denoising complexity of a 4D light field becomes $\mathrm{o}(kn_a^2 N^2)$, resulting in a total complexity of $\mathrm{o}\big((2m + kn_a^2)N^2\big)$. Similarly, we can analyze the computation complexity per iteration for compressive light field imaging methods based on the model in Supplementary Eq. 17 to 19. Supplementary Table 1 summarizes the characteristics of CLIP and compressive light field photography. It is worth noting that the computation complexity of Marwah's work does not account for the dictionary learning process, and the regularization is applied on the entire light field. Also, the convolution model of the diffuser-camera is accelerated by FFT.

Supplementary Table 1 Comparison of CLIP and compressive light field photography

Methods	Sensor	Light field size	Measurement data size	Compression axis	Computation complexity	
Ashok[13]	2D	$n_a \times n_a \times N \times N$	$r \times N \times N$	Angular or spatial	$\mathrm{o}\big((2r + k)n_a^2 N^2\big)$	
Babacan[14]	2D	$n_a \times n_a \times N \times N$	$r \times N \times N$	Angular	$\mathrm{o}\big((2r + k)n_a^2 N^2\big)$	
Marwah[5]	2D	$n_a \times n_a \times N \times N$	$N \times N$	Angular	$\mathrm{o}\big((2 + k)n_a^2 N^2\big)$	
Cai[7], Antipa[8]	2D	$n_a \times n_a \times N \times N$	$N \times N$	Angular	$\mathrm{o}\big((4logN + kn_a^2)N^2\big)$	
CLIP	0D, 1D, 2D	$n_a \times n_a \times N \times N$	m ($\leq N \times N$)	Angular and/or spatial	4D light field	$\mathrm{o}\big((2m + kn_a^2)N^2\big)$
					Refocus image	$\mathrm{o}\big((2m + 2n_a^2 + k)N^2\big)$

Supplementary Note 6. Generality of CLIP

While recovering a 4D light field is always under-determined in CLIP and compressive light field photography methods, directly recovering a refocused image by CLIP is not necessarily the same. As a result, CLIP isn't bounded to the compressive regime, though one of its major appeal is to record a large-scale light field with a highly limited sensor budget. When working in the compressive regime, it is important to evaluate whether the system matrix $\mathbf{A}'$ of CLIP supports a uniform recovery of arbitrary k-sparse vectors (vectors with at most k non-zero entries) in the classic sparse signal model by computing the restricted isometry property (RIP) of matrix $\mathbf{A}'$. However, RIP is not a necessary condition and computing the RIP constant is an NP-hard problem. Up to now, only a limited types of matrices have been proven to satisfy RIP with an exponentially high probability. On the other hand, it was shown[15] that there is an absence of RIP in a range of practical compressive imaging applications, and yet, experimental image recovery is excellent. These applications include compressive *x*-ray tomography, MRI, and single pixel cameras. The work of Bastounis[15] and Roman[16], among other similar works[17], attributed the correct recovery of

image **x** to the structured-sparsity of **x** (that is, the sparsity of **x** has a structure instead of exhibiting an arbitrary pattern), and together with an extended concept of RIP in levels, explained the success of these compressive imaging methods in practice, despite that their measurement matrices failed to satisfy the classic RIP. As natural images are highly structured, and CLIP with 0D and 1D sensors are transformed from the single pixel cameras and *x*-ray tomography methods respectively, it is expected that CLIP can attain similar imaging performance in practice.

We followed the philosophy of generalized flip test proposed by Roman et.al.[16] to evaluate the general applicability of CLIP under the structured-sparsity signal model. This idea of the test is to evaluate the reconstruction quality of different images with the same sparsity. To generate such images, we applied shift, flip, rotation operation on some image part, and evaluated the reconstruction error using normalized mean square errors (NMSE). As CLIP deals with light field data, these operations should be applied to 3D objects. To this end, the 3D scenes were modelled in Blender software for rendering the 4D light field data on a regular 2D grid.

Throughout the manuscript, synthetic CLIP measurement with 1D and 0D sensors were obtained as follows. In CLIP-0D, each sub-aperture image is encoded with random binary codes to yield $m_k=m/l$ single-pixel readings. For CLIP-1D, the measurements are obtained in three steps: a) generate m/N projection angles α uniformly in the range of [0, 180°]; b) randomly permute the angles α and distribute evenly into the l sub-apertures; c) calculate for each sub-aperture image the projection data along the assigned angles. The sampling ratio (SR) is defined as the quotient between the total number of measurements m and the image size N^2 (rather than the 4D light field). For this test, we fixed SR at 0.5.

Supplementary Figure 6 and 7 show the CLIP imaging results for two different scenes under various focus settings, with NMSE listed on Supplementary Table 2. It is noted that CLIP consistently yields a NMSE below 10% for SR=0.5, indicating its generality in coping with natural scenes when working in the compressive regime. Further results demonstrating the generality of CLIP are given in Supplementary Note 11, which employs CLIP (with different sampling ratio SRs) to represent experimentally acquired light field data for scenes with different BRDFs.

Supplementary Table 2. NMSE of CLIP reconstruction for synthetic scene 1 and 2

	s	CLIP-1D				CLIP-0D			
		Original	Shift	Flip	Rotate	Original	Shift	Flip	Rotate
Scene 1	-0.8	7.28%	8.75%	8.13%	7.26%	8.75%	9.66%	9.70%	9.36%
	-0.1	9.53%	7.10%	7.14%	7.59%	8.71%	7.97%	9.22%	8.18%
	0.5	7.22%	7.96%	7.17%	6.38%	8.97%	9.34%	8.52%	8.39%
Scene 2	-0.6	4.71%	4.34%	4.88%	4.54%	5.40%	5.37%	5.20%	4.64%
	0	5.52%	3.70%	3.67%	4.28%	5.47%	5.57%	5.54%	5.54%
	0.8	6.48%	4.21%	4.40%	4.89%	5.22%	4.86%	4.59%	5.08%

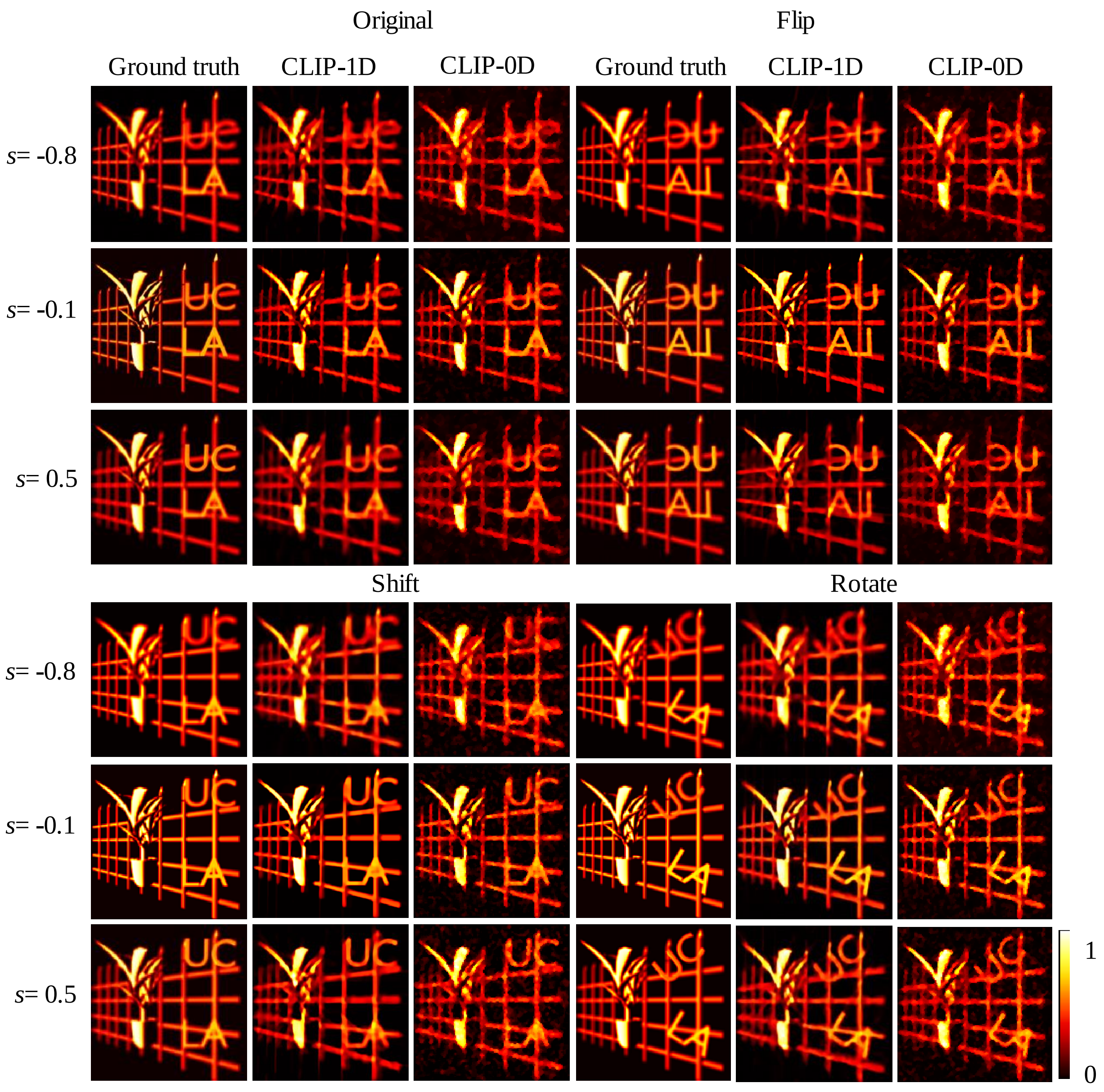

Supplementary Figure 6. Generalized flip test of CLIP reconstruction for synthetic scene 1 with SR = 0.5. The ground truth light field size is 8×8×128×128, and the measurement data size is 64×128, leading to a data reduction of 128. *s*: refocusing parameter, CLIP: compact light field photography.

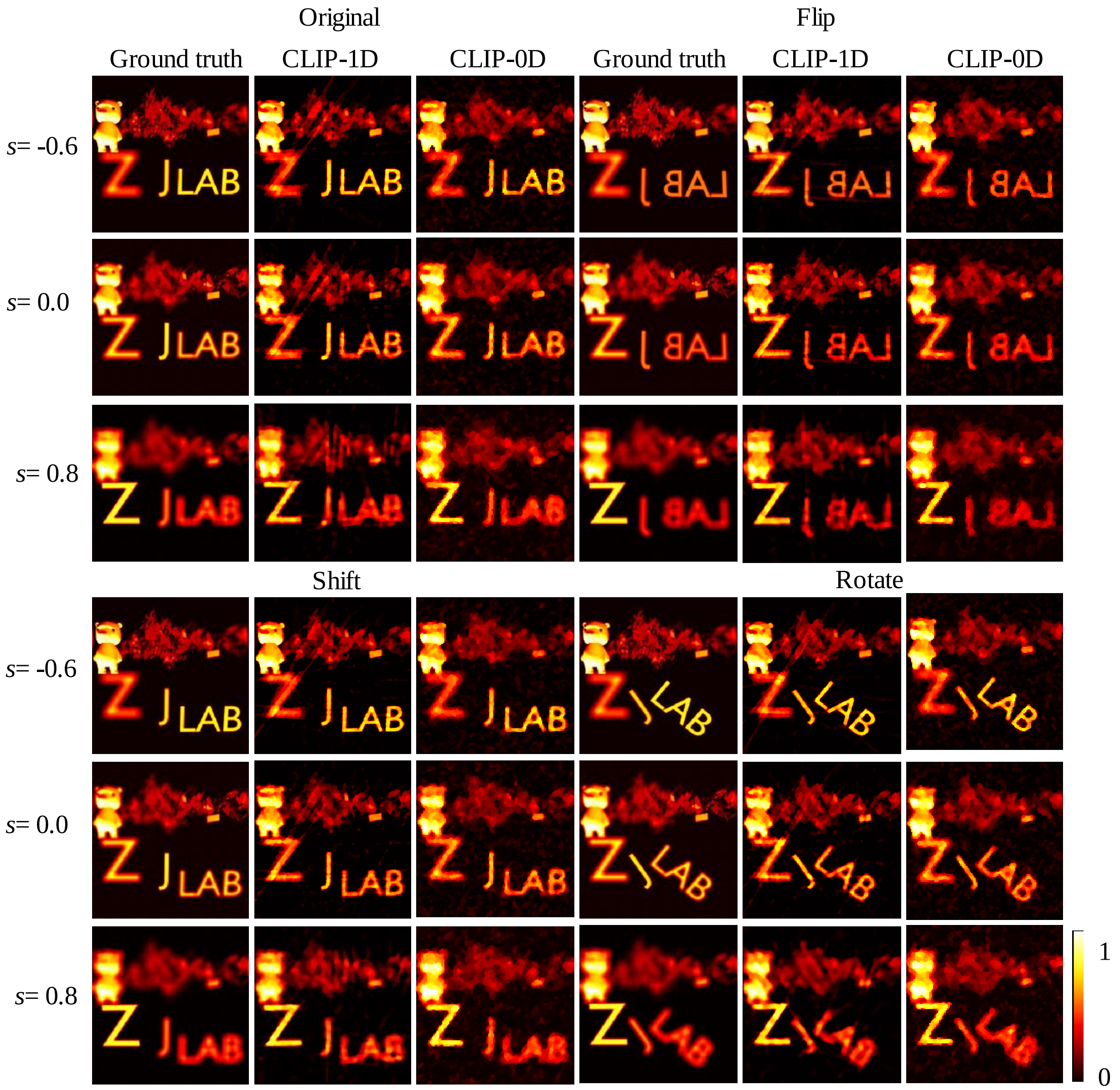

Supplementary Figure 7. Generalized flip test of CLIP reconstruction for synthetic scene 2 with SR = 0.5. The ground truth light field size is 8×8×128×128, and the measurement data size is 64×128. *s*: refocusing parameter, CLIP: compact light field photography.

Supplementary Note 7. Quantitative evaluation of CLIP performance in experiments

We quantitatively evaluated the performance of CLIP via experimental measurements when feasible and turned to synthetic studies otherwise. This is because for computational imaging employing nonlocal sampling strategies, ground truth data is typically difficult to obtain experimentally: a system reconfiguration with perfect alignment is necessary. Taking CLIP imaging with 1D sensors for example, one needs to swap the cylindrical lenslet array into its

spherical counterpart and adds a 1D scanning to obtain the ground-truth light field. This reference imaging needs to be precisely realigned to show the same magnification and field of view with CLIP: any mismatch will otherwise bias the quantitative evaluation of its imaging accuracy.

For CLIP imaging with 0D sensors, the 4D light field can be fully sampled (though not based on conventional 2D sensors): for each angular position behind the lens, the sub-aperture image can be acquired with a measurement number equal to or larger than the image resolution (thus doesn't rely on compressive sensing), and this imaging process is repeated at all angular positions. CLIP measurement can be readily obtained from this dataset by extracting a small subset measurement from each angular position and stacking the complementarily extracted data into a final measurement as described by Supplementary Eq. 20. We present experimental validation of CLIP with 0D sensor in this section and synthetic evaluation of CLIP with 1D sensor in the following sections.

Two different scenes composed of printed letters were imaged by CLIP-0D experimentally, and both the 4D light field and direct image reconstructions are demonstrated under different sampling ratio SR. The ground truth 4D light field has a resolution of 4×4×128×128 and was obtained by reconstruct each sub-aperture image using a complete measurement. Similarly, the ground truth refocused image was obtained from the 4D light field. Supplementary Figure 8 and 9 shows the 4D light field reconstruction results by CLIP for the two scenes and the direct reconstruction of different refocused images are given in Supplementary Figure 10. The reconstruction error is quantified by NMSE in Supplementary Table 3. It is noted that both the 4D light field and direct reconstruction of refocused images attained a NMSE error below 10% in experiments.

Supplementary Table 3. NMSE of CLIP-0D reconstruction for experimental scenes

	Sampling ratio	4D light field	s = -1	s = -0.3	s = 0.3	s = 1
Scene 1	SR = *1*	7.08%	1.94%	1.53%	1.39%	1.76%
	SR = *0.5*	7.31%	2.32%	2.15%	1.83%	2.29%
	SR = *0.25*	8.84%	5.41%	3.06%	2.57%	3.22%
Scene 2	SR = *1*	1.34%	0.94%	0.66%	0.61%	0.67%
	SR = *0.5*	2.13%	1.34%	1.39%	1.35%	1.46%
	SR = *0.25*	5.06%	3.99%	4.16%	3.75%	3.07%

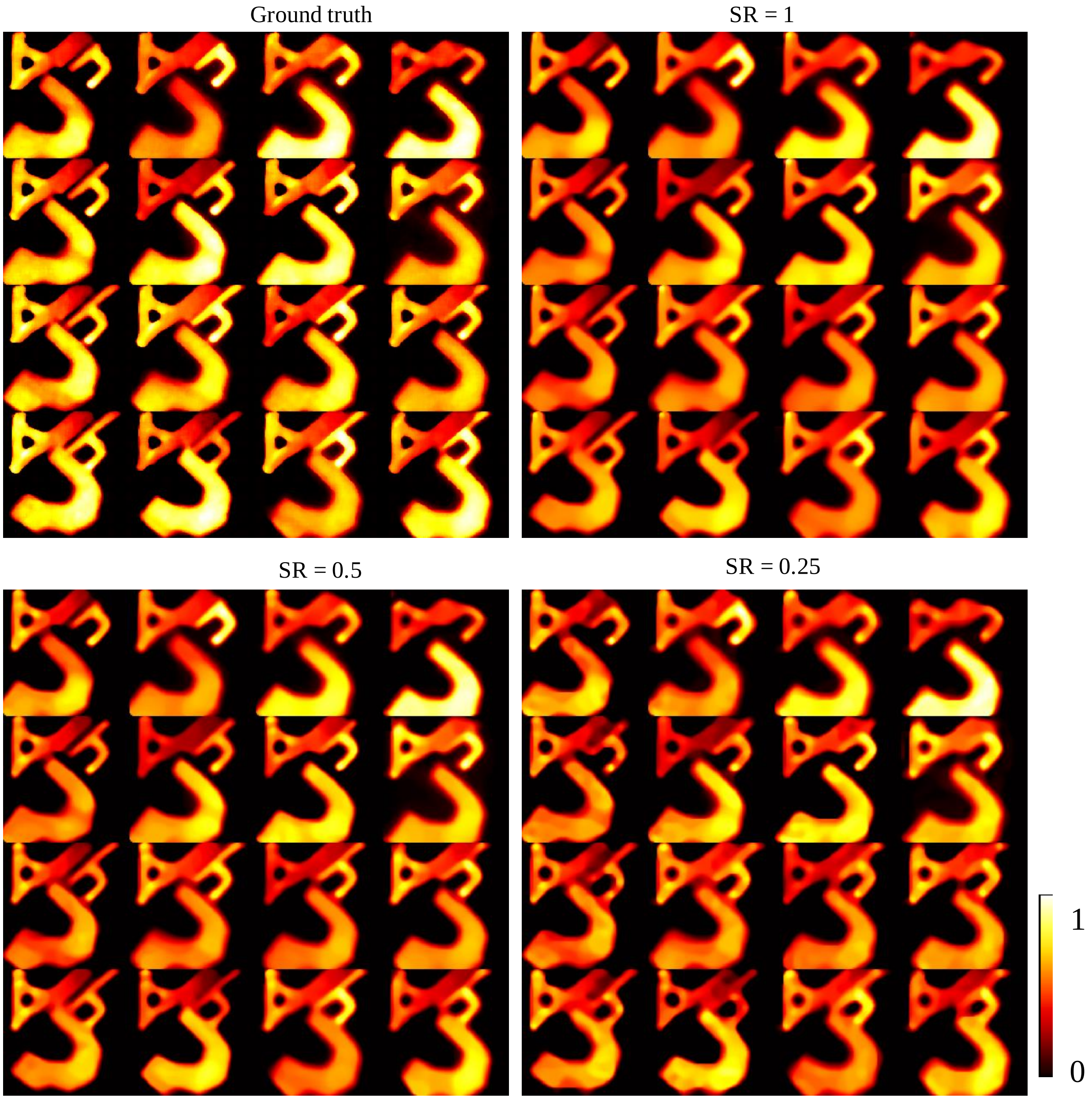

Supplementary Figure 8. CLIP-0D 4D light field reconstruction for experimental scene 1. The sampling ratio (SR) is varied from SR = 1 to 0.25.

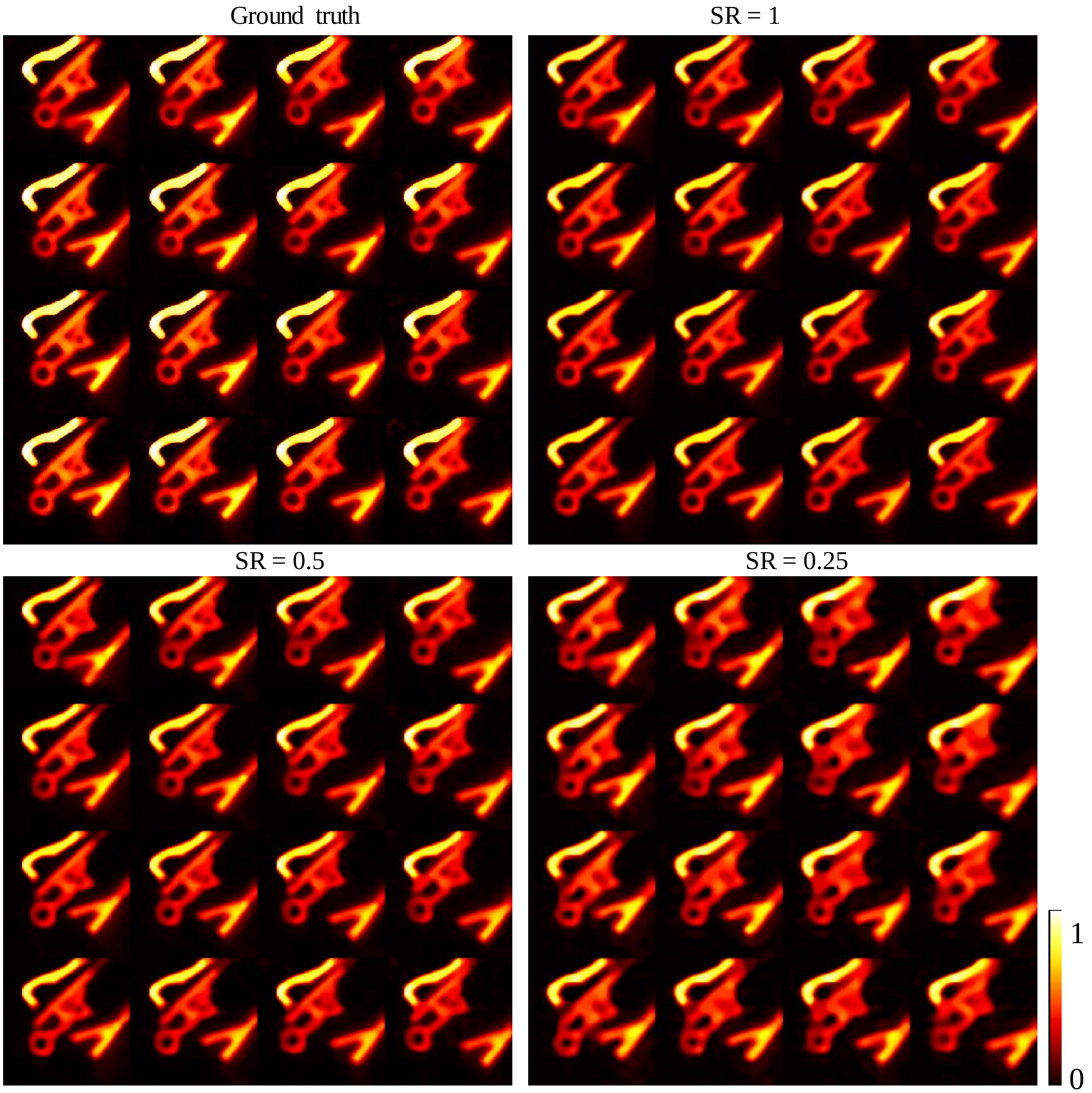

Supplementary Figure 9. CLIP-0D 4D light field reconstruction for experimental scene 2 The sampling ratio (SR) of CLIP is varied from SR = 1 to 0.25. SR: sampling ratio.

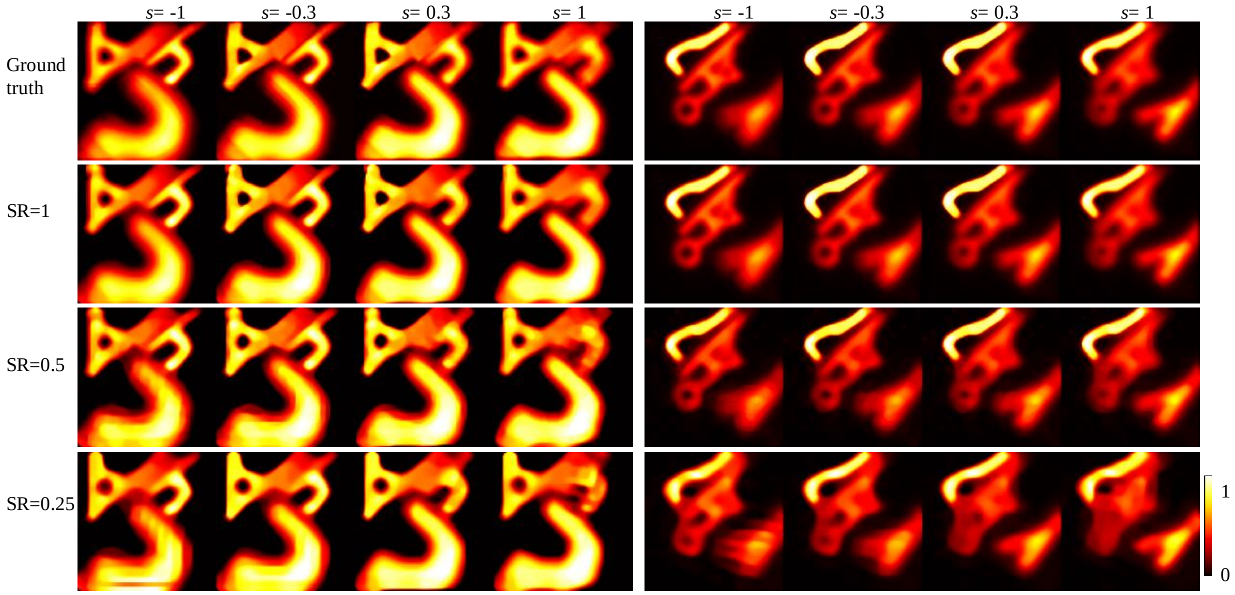

Supplementary Figure 10. CLIP-0D direct reconstruction of refocused images for experimental scene 1 and 2. The sampling ratio (SR) of CLIP is varied from SR= 1 to 0.25. *s*: refocusing parameter.

Supplementary Note 8. CLIP 4D light field reconstruction versus direct reconstruction

While CLIP can recover a 4D light field as demonstrated in previous note, we show here that directly recovering a refocused image can better accommodate complex scenes, particularly for imaging with lower dimension (1D or 0D) sensors. Marwah's work relied on a dictionary learning process to obtain a representation basis to better sparsify the 4D light field, thereby attaining excellent 4D light field reconstruction for complex scenes. On the other hand, Antipa[7] pointed out that improper regularization of the 4D light field in diffuser-based camera can degrade (or even destroy) the angular information in the light field.

In contrast, CLIP doesn't rely on high quality 4D light field reconstruction to obtain excellent refocused images: CLIP's complementary measurements among sub-apertures can significantly improve the refocused images despite the recovered 4D light field may not be of high quality, which is the case unde the compressive regime. Further, CLIP can directly recover a refocused image like coded-aperture and wavefront-coding methods to accommodate complex scenes better, as explained in previous section. We demonstrate this via a synthetic study for the synthetic scene 2 and an experimentally acquired light field from the 'letter scene', using a sampling ration of SR=1. During the reconstruction for the 4D light field, the regularization parameter is tuned from to obtain a best refocused image from the light field data. Supplementary Figure 11 shows the recovered 4D light field and refocused images for the two scenes under the CLIP-1D (a and b) and CLIP-0D (c and d) implementations, with the NMSE listed in Supplementary Table 4. It is noted that while the light field suffers from significant background signals and noises, the refocusing processing coherently assembles CLIP's complementary imaging across the sub-apertures to yield substantially better refocused image. Moreover, CLIP's direct reconstruction further improved the quality of the refocused image by rendering more image details and a higher contrast.

Supplementary Table 4. NMSE of 4D and direct CLIP reconstructions

	Scene	Method	*s*=-1.0	*s*=1.0		Method	*s*=1.0	*s*=1.0
CLIP-1D	1	4D recon.	11.98%	6.08%	CLIP-0D	4D recon.	10.47%	10.67%
		Direct recon.	3.66%	4.16%		Direct recon.	6.23%	7.35%
	2	4D recon.	18.68%	8.48 %		4D recon.	13.02%	7.53%
		Direct recon.	6.33%	6.75%		Direct recon.	4.41%	4.05%

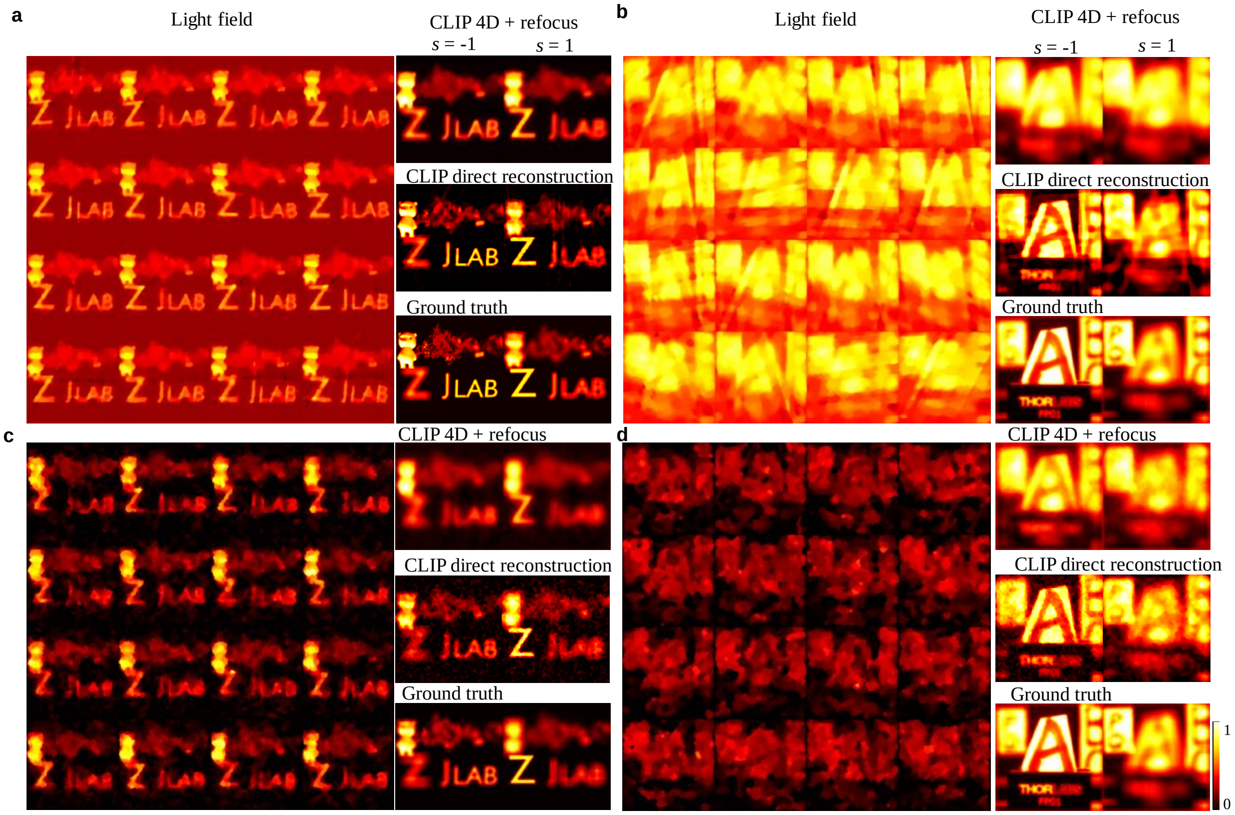

Supplementary Figure 11. 4D light field reconstruction versus direct reconstruction of refocused images by CLIP. a-b, CLIP-1D reconstruction for the synthetic scene and the experimental 'letter' scene. c-d, CLIP-0D reconstruction for the two scenes. The sampling ratio (SR) of CLIP is fixed at SR=1. CLIP: compact light field photography; *s*: refocusing parameter.

Supplementary Note 9. CLIP robustness

The robustness against missing pixels of CLIP is demonstrated to some extent by imaging with sparse 2D detectors in Supplementary Note 5. Here, we test the robustness of CLIP against erroneous measurements. Two typical errors are dead (or missing) pixels and saturated sensor readings. We tested the case that the measurement containing both types of errors by first

normalizing the measurement data, and then randomly setting part of the measurement to 0 (dead) or 1 (saturated). The error induced by defective measurement is evaluated by NMSE for both the raw measurement data and reconstructed images. Fixing the sampling ratio SR at 1, we varied the percentage of the erroneous measurement from 0.1% to 1% for the experimental data in CLIP-0D, and 1% to 10% for the synthetic data in CLIP-1D. Supplementary Figure 12 shows the CLIP imaging results, and the corresponding NMSEs are summarized in Supplementary Table 5. Owing the nonlocal data acquisition strategy and the regularization step, the reconstructed image error in CLIP is substantially smaller than the error in the raw measurements, making it more robust than classic imaging methods.

Supplementary Table 5. NMSE of CLIP reconstruction with erroneous measurement

CLIP-0D					CLIP-1D				
Error percent		0.1%	0.2%	1%	Error percent		1%	5%	10%
Exp. Scene 1	Data error	5.68%	9.9%	37.2%	Synthetic Scene 1	Data error	14.53%	47.90%	66.39%
	Image error	1.54%	2.08%	11.24%		Image error	5.07%	12.1%	15.2%
Exp. Scene 2	Data error	22.52%	36.29%	75.88%	Synthetic Scene 2	Data error	8.99%	34.83%	53.43%
	Image error	4.50%	4.7%	6.5%		Image error	1.68%	6.05%	9.38%

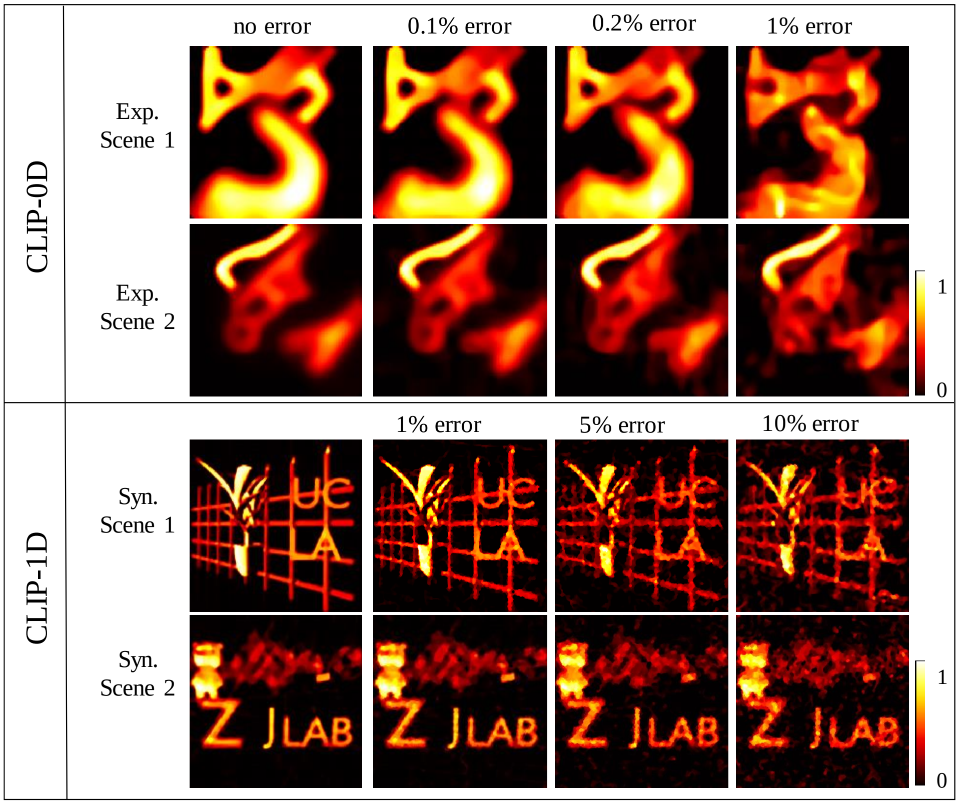

Supplementary Figure 12. CLIP reconstruction with different amount of erroneous measurement data. The error in the title is listed as percentage of the total measurement number. Syn.: synthetic; Exp.: experimental; CLIP: compact light field photography.

The robustness of CLIP for photon-starved imaging applications, which are limited by Poisson (or shot) noises, are demonstrated in Supplementary Figure 13 by varying the maximum number of photons in measurement from 400 to 10000. As indicated by the NMSE in Supplementary Table 6, while CLIP-0D is more susceptible to Poisson noises, it can still recover the rough structure of complex scenes with a maximum of only 1000 photons. Since single pixel imaging usually benefit from a larger photon-detector, CLIP-0D is expected to cope well with shot-noise limited imaging applications.

Supplementary Table 6. NMSE of CLIP imaging with different number photons

	Photons	Scene 1	Scene 2		Photons	Scene 1	Scene 2
CLIP-1D	400	7.30%	2.85%	CLIP-0D	400	57.88%	43.45%
	1000	6.54%	2.66%		1000	40.62%	25.45%
	4000	5.92%	2.55%		4000	20.58%	16.16%
	10000	6.01%	2.53%		10000	14.95%	7.05%

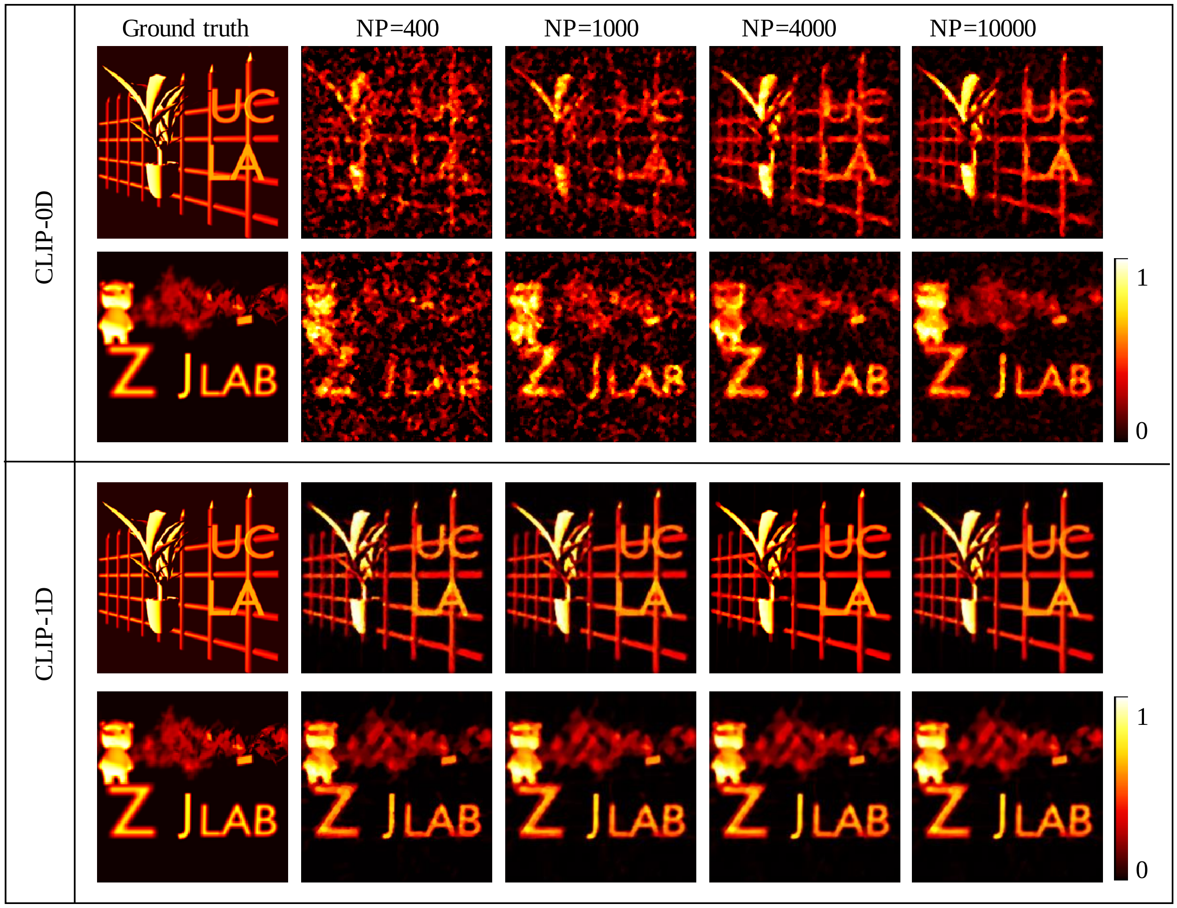

Supplementary Figure 13. CLIP reconstruction with different number of photons. NP is the maximum number of photons in the measurement dataset. CLIP: compact light field photography.

Supplementary Note 10. 3D Imaging through occlusion by ToF-CLIP

Our proof of concept demonstration of seeing through occlusion used a oblique illumination to ensure both the occluder and the obscured objects are covered by the laser. In practice, high power lasers are bulky and most LiDAR systems used an array of synchronized laser diodes to deliver sufficient energy within a flash illumination. For ToF-CLIP under a camera array implementation, it is feasible to distribute the laser diodes to each invidual view as illustrated in Supplementary Fig. 14a, where the laser diodes (could be more than one) in each view provide a diverged illumination and are closely packed with the sensor to make them roughly co-located. This allows the laser diodes and time-of-flight sensor to share approximately the same field of view and therefore facilitates imaging through occlusions without special coordination of the illumination.

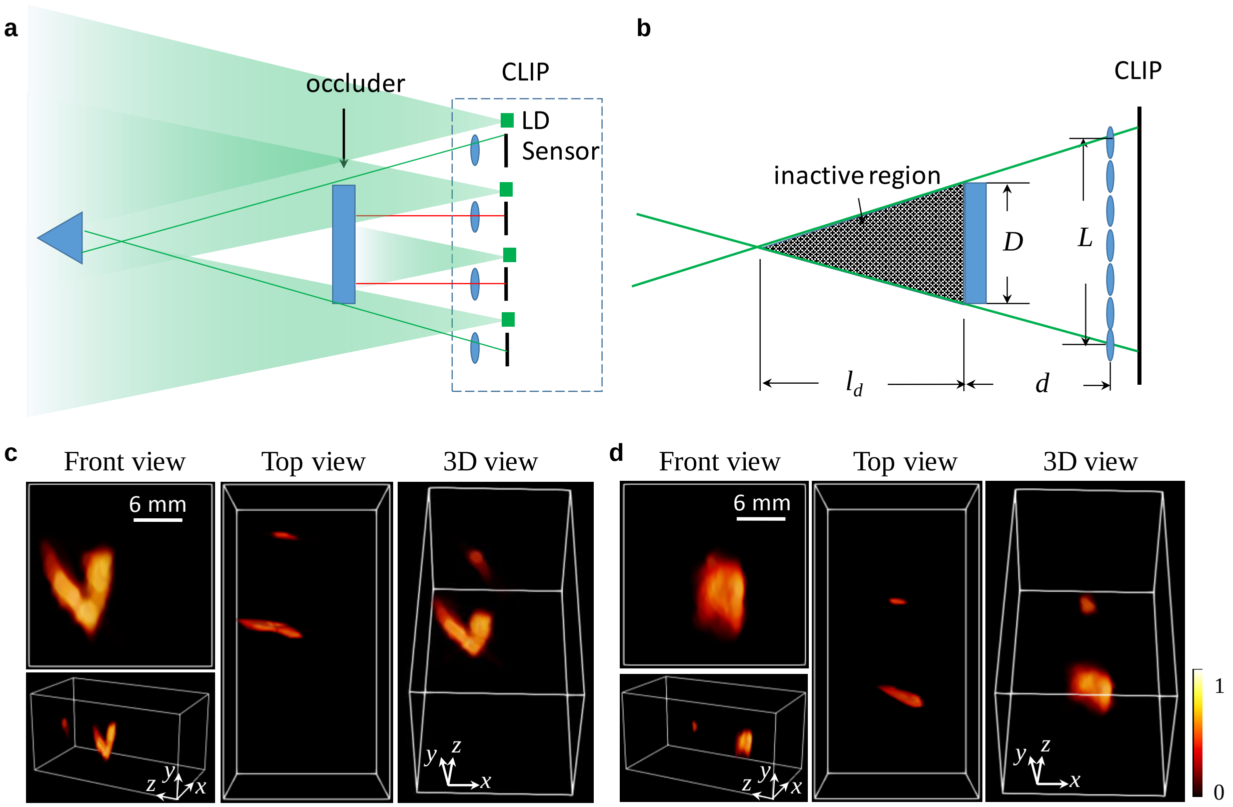

Supplementary Figure 14. 3D imaging through occlusions. a ToF-CLIP camera with roughly con-located laser diodes and sensor for seeing thourgh occlusions. **b** Geometry for calculating the inactive region caused by the occluder, and the experimental setup for dynamic imaging studies. **c-d** Additional imaging results of seeing through occlusions. The reconstructed 3D images are rendered in different perspectives. A small circular plate are blocked by a letter V (c) and a triangular plate is placed behind a rectangular plate (d). LD: laser diode; CLIP: compact light field photography; 3D: three-dimensional.

The ability to see through occlusions relies critically on part of the object being visible to at least one view of the ToF-CLIP camera. As a result, there will be an inactive region where the object cannot be detected if an occluder blocks its signal in all the views. Supplementary Figure 14b shows the geometry for determining the inactive region caused by a solid occluder (i.e., no

hollow structures allowing some light to pass through). Denoting the aperture base line of the CLIP camera as L, the occluder size and its distance to the camera as D and d respectively, the length of the inactive region can be calculated as $l_d = dD/(L - D)$. A negative l_d ($D>L$) indicates the inactive region extends to infinity and thus it will not be possible to sense any objects behind the occluder. This is common in conventional imaging approaches, where the camera aperture has a small baseline L.

A photograph of system setup for the dynamic imaging experiment is shown in the bottom of Supplementary Figure 14b, where the camera baseline L is ~15 mm, and the occluder was placed at approximately d=50 mm (or ~40 mm in the static studies) from the lenslet array. For an occluder with width $D \approx 6$ (or 10) mm, the inactive region is hence $l_d \approx 33\ (or\ 80) mm$. The object was positioned at a distance ~70 mm (or >90 mm for different static studies) from the occluder to avoid falling into the inactive region. Two additional experimental results of 3D imaging through occlusions are shown in Supplementary Fig. 14c-d, where the obscured objects are placed at a distance behind the occluder to be outside the inactive region. Similar to the large camera array system[18], CLIP's reconstruction process essentially synthesize the small apertures from all the views into a large one, and hence allow it to peek through occlusions. A key difference is that while the conventional camera array can directly visualize different parts of the obstructed object from certain views, CLIP only captures an implicit nonlocal measurement for it.

We further compare CLIP with conventional light field imaging for seeing through occlusions via synthetic studies. The 4D light field for 3D scenes were rendered in Blender software with a resolution of 8×8×128×128, and CLIP measurement were obtained as in previously sections. Unlike ToF based measurements that can separate signals of the occluder and occluded objects in time, conventional imaging systems can only defocus the occluder, yielding significant background for visualizing the occluded objects. To emulate ToF measurement for minimizing background, the occluder can be made black in Blender such that its image signal is negligible in the generated light field. Supplementary Figure 15 shows four examples of imaging through occlusions: a mannequin standing behind a tree, the mannequin partially occluded by the black rectangular plate, the 'CLIP' letter placed behind a bush, and the 'CLIP' letter being blocked by a black rectangular occluder. The CLIP reconstruction NMSE errors are shown in Supplementary Table 7. It is noted that even with a sampling ratio of SR=0.5 that corresponds to a reduction of the 4D light field by 128 times, CLIP can effectively see through severe occlusions with an error below 10%. With ToF measurement that produces far sparser 2D instantaneous images and separate the occluder signal in time, as emulated by black occluder, CLIP can hence attain background-free imaging of occluded objects with a small number of sensors.

Supplementary Table 7. NMSE of CLIP imaging through occlusions

	Scene	SR=1.0	SR=0.5		Scene	SR=1.0	SR=0.5
CLIP-1D	1	2.14%	2.68%	CLIP-0D	1	4.29%	6.96%
	2	0.89%	2.44%		2	2.19%	3.810%
	3	3.03%	4.83%		3	4.37%	6.41%
	4	2.62%	4.12%		4	3.25%	4.77%

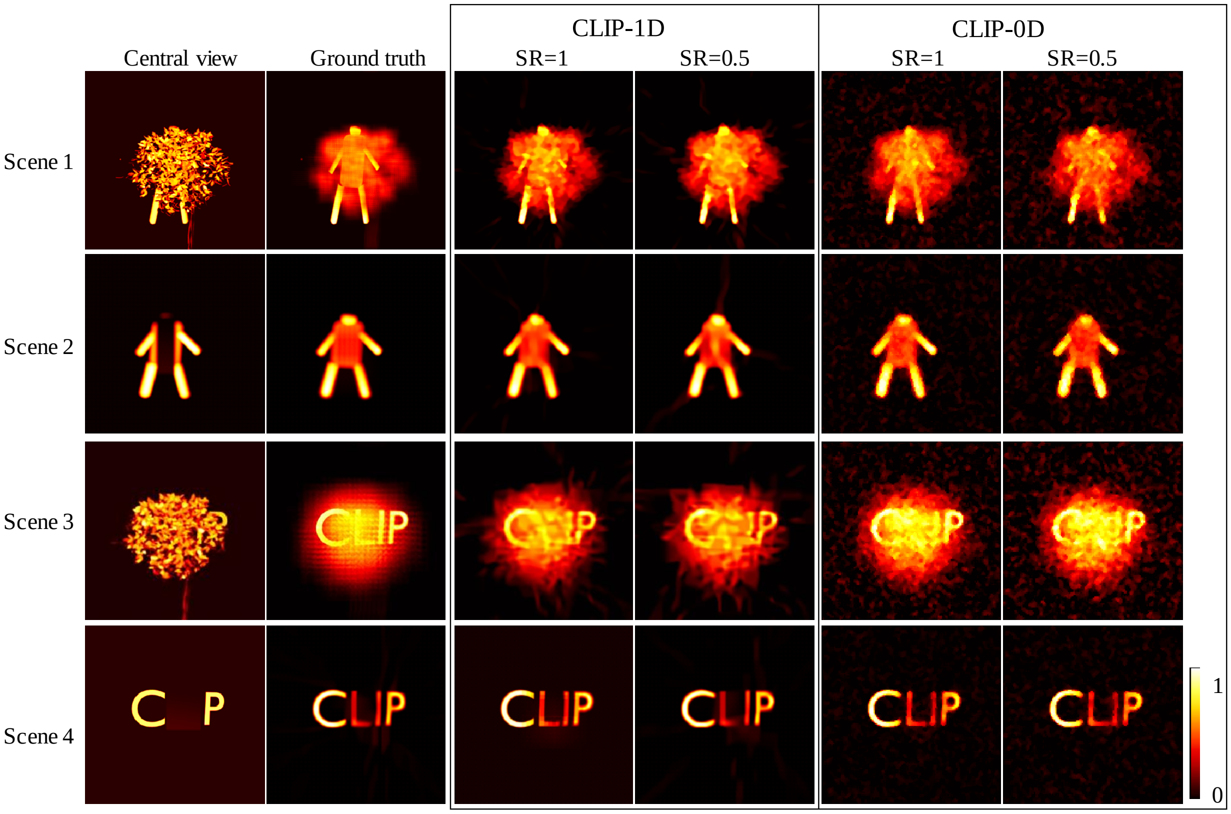

Supplementary Figure 15. CLIP imaging through occlusions for four different scenes. The sampling ratio (SR) of CLIP is varied from SR= 1 to 0.5. CLIP: compact light field photography.

Supplementary Note 11. CLIP generality: representing 4D light field data

We show additional ressults of applying CLIP to represent 4D light field acquired by a custom unfocused plenoptic camera for scenes with different BRDFs. The ground truth light fields have a dimension of l=8×8 (angular) and N^2=128×128 (spatial), and the CLIP measurement are generated similarly as the synthetic study in Supplementary Note 5.

Supplementary Figure 16 shows the CLIP representation of the light field data of the 'letters' scene composed of three-letter plates separated at different depths. The ground truth images generated by canonical processing of the 4D light fields, and the corresponding results reproduced by the CLIP framework are arranged in a tabular format. For both the CLIP-0D and CLIP-1D approaches, CLIP achieved the same light field processing capabilities as conventional light field cameras, including post-capture refocusing, depth retrieval, and extending the depth of field. The recovered image quality in CLIP varies with the sampling ratio SR: a small oversampling with SR=2 leads to almost indistinguishable imaging results from the ground truth. At the same time, compressive sensing (SR<1) tends to degrade the image resolution by washing out high-frequency features, as a stronger image prior is imposed herein for compressive reconstruction. Still, we emphasize that the measurement number is only a fraction of that in a single sub-aperture image

and is two orders of magnitude less than the full 4D light field. The NMSE of the CLIP representation results are summarized in Supplementary Table 8.

Supplementary Table 8. NMSE of CLIP representation of the 'letters' scene

	SR	2	1	0.5		SR	2	1	0.5	0.25
CLIP -1D	*s* = -1.0	1.75%	2.79%	5.32%	CLIP -0D	*s*= -1.0	1.75%	2.60%	3.99%	6.61%
	s = -0.4	1.47%	2.63%	4.54%		*s*= -0.4	1.48%	2.49%	4.06%	5.58%
	s = -0.1	1.59%	3.04%	5.91%		*s*= -0.1	1.64 %	2.84%	4.48%	7.50%

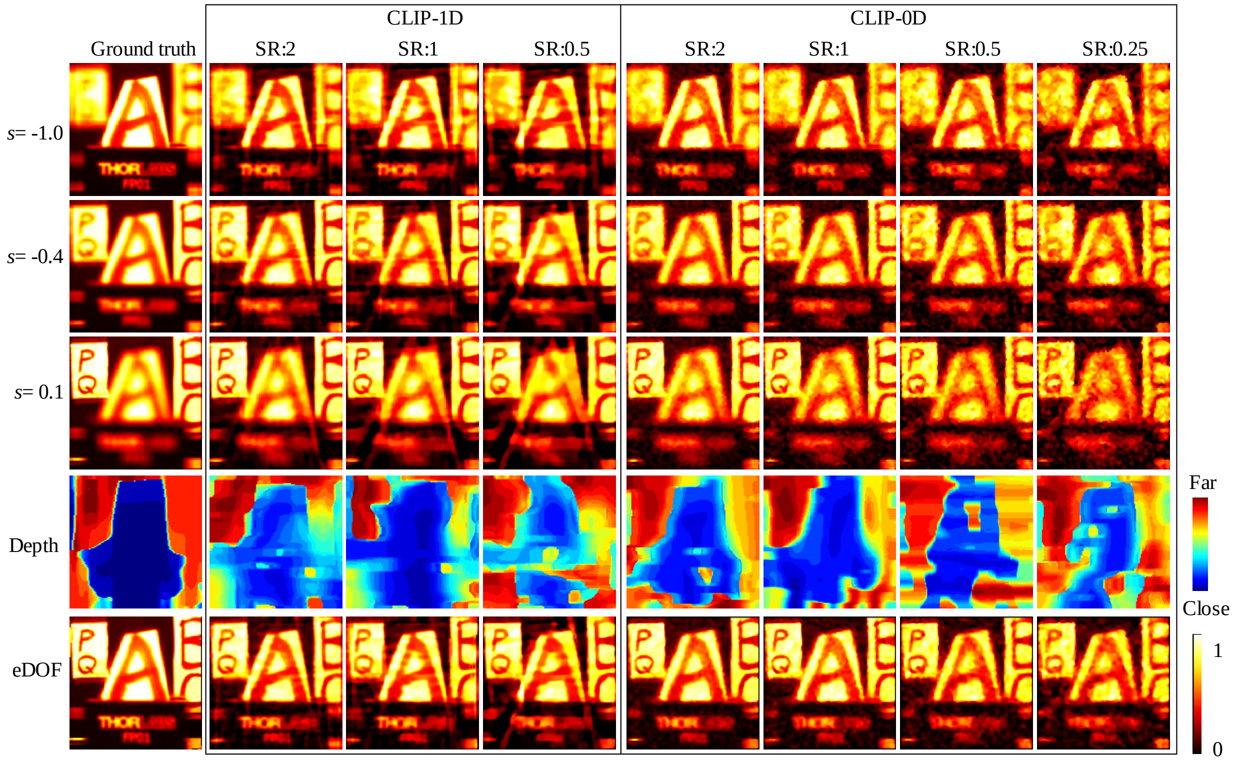

Supplementary Figure 16. CLIP representation of light fields for the 'letters' scene. Sampling ratio SR=1 indicates the measurement number m equals the image size: $m=N\times N$. LIFT: light field tomography; SPC: single-pixel camera. The refocusing capability is rendered ($s<0$ refocus to close depth) when sweeping the imaging focus from close to far, gradually bringing three-letter plates into focus. Although CLIP reconstruction becomes nosier and leads to more deficient depth maps as the sampling ratio decreases, the extracted depth map can correctly separate the three letter plates into different depths in all cases. eDOF: extended depth of field; CLIP: compact light field photography; *s*: refocusing parameter.

The images in Supplementary Fig. 17-18 are taken under the macro photography setup with a imaging magnification around 0.5. While the assumption of uniform angular intensity might become less accurate in this setting, CLIP managed to reproduce light field imaging results comparable with those yielded by convetional methods, even for the 'bolt-letter' scene that contain a shinny metal bolt. This illustrates that for applications where quantititavie analysis based on image intensity is not critical, CLIP can be employed for efficient light field imaging. Also, although the depth maps become noiser as the samping ratio SR gets smaller, the relative depth

for regions containing sufficient textures (e.g. the letters part) can be recovered in all the cases. Supplementary Table 9 and 10 detailed the NMSE of the CLIP representation for the two scenes.

Supplementary Table 9. NMSE of CLIP representation of the ‘slanted-text’ scene

	SR	2	1	0.5		SR	2	1	0.5	0.25
CLIP -1D	*s* = -1.0	0.97%	4.5%	9.97 %	CLIP -0D	*s* = -1.0	3.01%	4.91%	8.03%	11.94%
	s = -0.4	0.80 %	1.78%	6.41%		*s* = -0.4	4.10%	3.85%	6.13%	9.31%
	s = 0.3	0.72%	3.39%	5.54%		*s* = 0.3	3.29%	5.88%	7.62%	15.77%

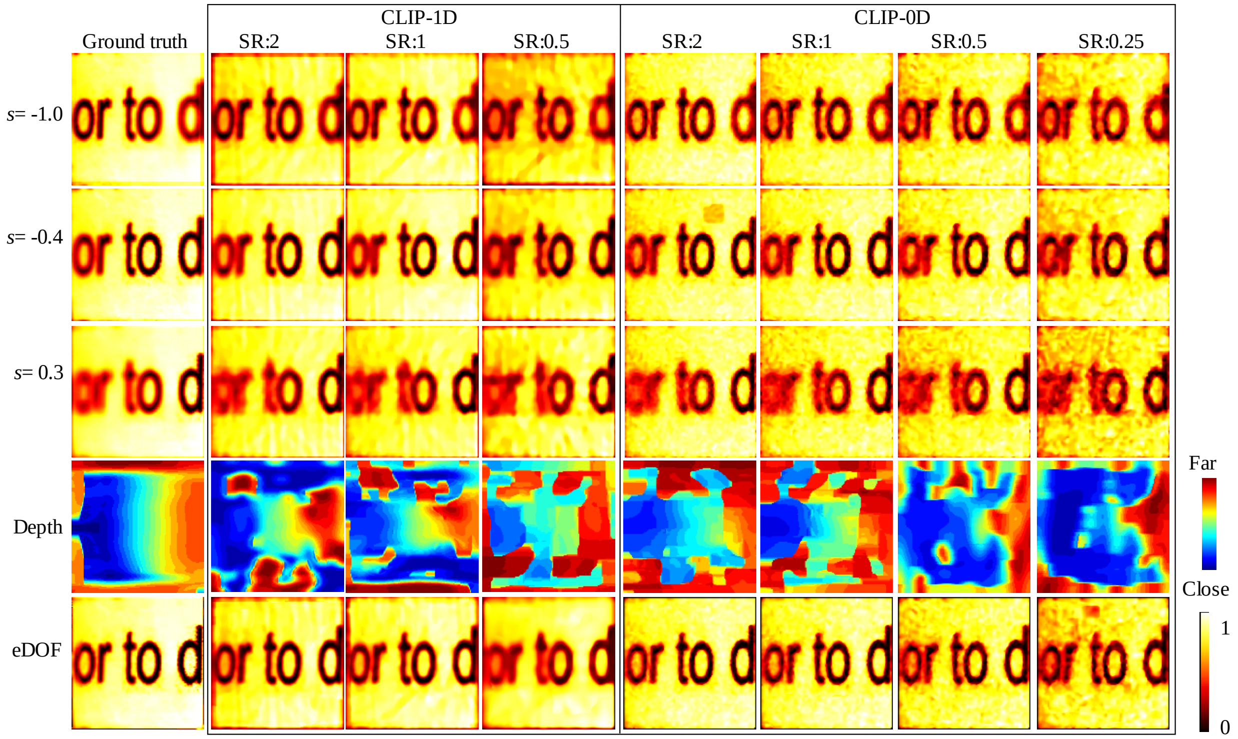

Supplementary Figure 17. CLIP representation of light fields for the ‘slanted-text’ scene. A paper printed with letters is attached on a slanted plate. Owing to the simplicity of the scene, an SR of 0.25 that leads to a reduction of light field data by 256 folds can be used for CLIP. eDOF: extended depth of field; CLIP: compact light field photography; *s*: refocusing parameter; SR: sampling ratio.

Supplementary Table 10. NMSE of CLIP representation of the ‘bolt-letter’ scene

s	SR	2	1	0.5		SR	2	1	0.5	0.25
CLIP -1D	*s* = -1.0	0.97%	2.46%	4.77 %	CLIP -0D	*s* = -1.0	0.82%	1.27%	1.96%	3.28%
	s = -0.5	0.80 %	2.04%	3.84%		*s* = -0.5	1.31%	1.72%	2.35%	3.25%
	s = 0.2	0.72%	1.70%	3.23%		*s* = 0.2	0.85%	1.22%	2.11%	2.94%

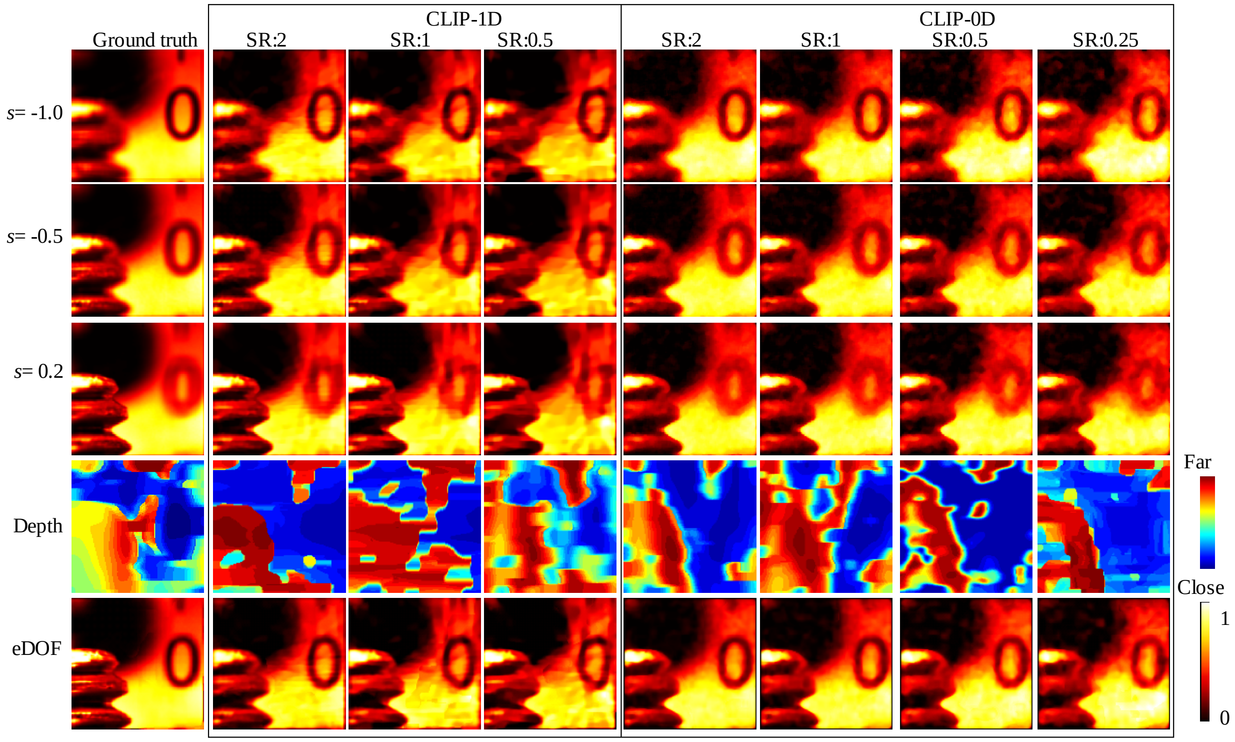

sSupplementary Figure 18. CLIP representation of light fields for the 'bolt-letter' scene. Note that the shinny regions on the metal bolt can be well recovered. eDOF: extended depth of field; CLIP: compact light field photography; *s*: refocusing parameter; SR: sampling ratio.

Supplementary Note 12. Hybrid frequency-time NLOS reconstruction for curved surfaces

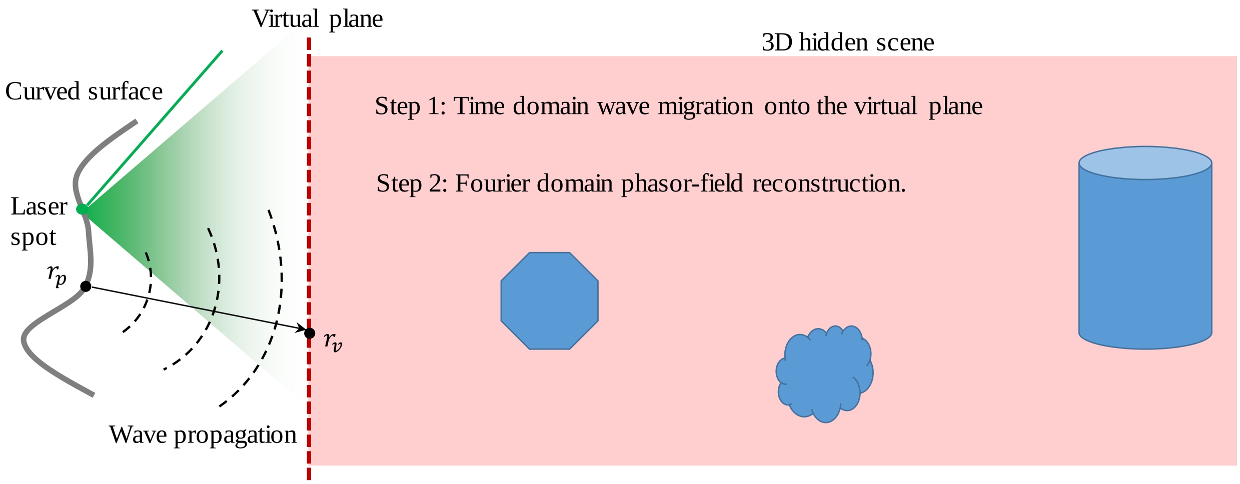

Supplementary Figure 19. Hybrid frequency-time domain reconstruction algorithm for NLOS imaging. A virtual planar surface between the curved surface the hidden 3D scene is chosen, and the measured spatiotemporal waveform on the curved surface is computationally propagated in the time domain onto the virtual planar surface. The frequency-domain solver[19] proposed by Liu. et.al can then be

readily exploited for recovering the hidden scene. Note that the virtual planar plane can span a larger baseline than that of the curved surface to reconstruct beyond the surface area, which is generally done by zero-padding the Fourier domain data in the frequency domain algorithms. r_p: laser spot; r_v: spot on the virtual plane.

Supplementary Note 13. Adjoint CLIP reconstruction for NLOS imaging

With real-time NLOS reconstruction being addressed by frequency domain algorithms[19–21], the CLIP image recovery should be similarly efficient for real-time acquisition of time-of-flight data. As the relaying wall is effectively the aperture in NLOS imaging, it is possible to tolerate some noises and artefacts on it while still obtain a recognizable reconstruction of the hidden objects. Though sub-optimal in terms of noise robustness, it could be useful for high-speed detection and tracking applications in the field, where imaging speed rather than quality is critical. In this case, we show that a fast CLIP recovery is possible by applying the adjoint operator of the forward model on the measurement data:

$$\tilde{\mathbf{h}} = [\mathbf{F}(\mathbf{d})]^{\mathbf{T}}\mathbf{f} = [\mathbf{F}(\mathbf{d})]^{\mathbf{T}}[\mathbf{F}(\mathbf{d})\mathbf{h} + \boldsymbol{\sigma}] = [\mathbf{F}(\mathbf{d})]^{\mathbf{T}}\mathbf{F}(\mathbf{d})\mathbf{h} + \boldsymbol{\sigma}', \quad (22)$$

where $\boldsymbol{\sigma}'$ is the amplified noises. The reconstruction is only accurate when the forward models $\mathbf{F}(\mathbf{d})$ is unitary, i.e., $[\mathbf{F}(\mathbf{d})]^{\mathbf{T}}\mathbf{F}(\mathbf{d}) = \mathbf{I}$, which is ususally not the case. Nevertheless, inverting linear systems with adjoint operators for an approximate solution has long been used in seismic imaging and computed tomography. With the image at each time being recovered independently, the computation complexity for the adjoint reconstruction of a time-of-flight data cube with a dimension of (N, N, N_t) is $o(N_tN^2m)$.

Supplementary Figure 20a-c show our experimental NLOS imaging results (maximum intensity projection along depth direction) for the three hidden objects using the iterative (left) and adjoint (middle) methods to recover the time-of-flight data. Owing to the sub-optimal reconstruction and low signal-to-noise ratio of the experimental data, the adjoint method leads to noiser NLOS images with strong background around the objects. However, the rough shape of the objects is still discernable and could be enhanced by a hard image thresholding in the right column. When the signal-to-noise ratio is reasonably large in the raw signal, the simple adjoint method can lead to high quality NLOS reconstruction as shown in the synthetic results in Supplementary Fig. 20d-f. The experimental 'office-scene' dataset[22] used there were acquired by a SPAD with an exposure time varied from 20 ms to 5 ms and 1 ms, and the maximum photon counts across the temporal data cube is 19, 15, and 6 respectively. To simulate NLOS imaging via CLIP, the time-of-flight data is transformed by the forward operator **F(d)** (for a fixed d) and then recovered with the adjoint method. With a sampling ratio of SR=*0.1* for the tomographic CLIP measurement, the NLOS image obtained by the adjoint method is very close to the ground truth for the dataset acquired with an exposure time of 20 ms. The rough shape of the scene can also be inferred most of the time, except for the case that emulates the CLIP acquisition of the dataset with an exposure time of 1 ms at sampling ratio of SR=0.05.

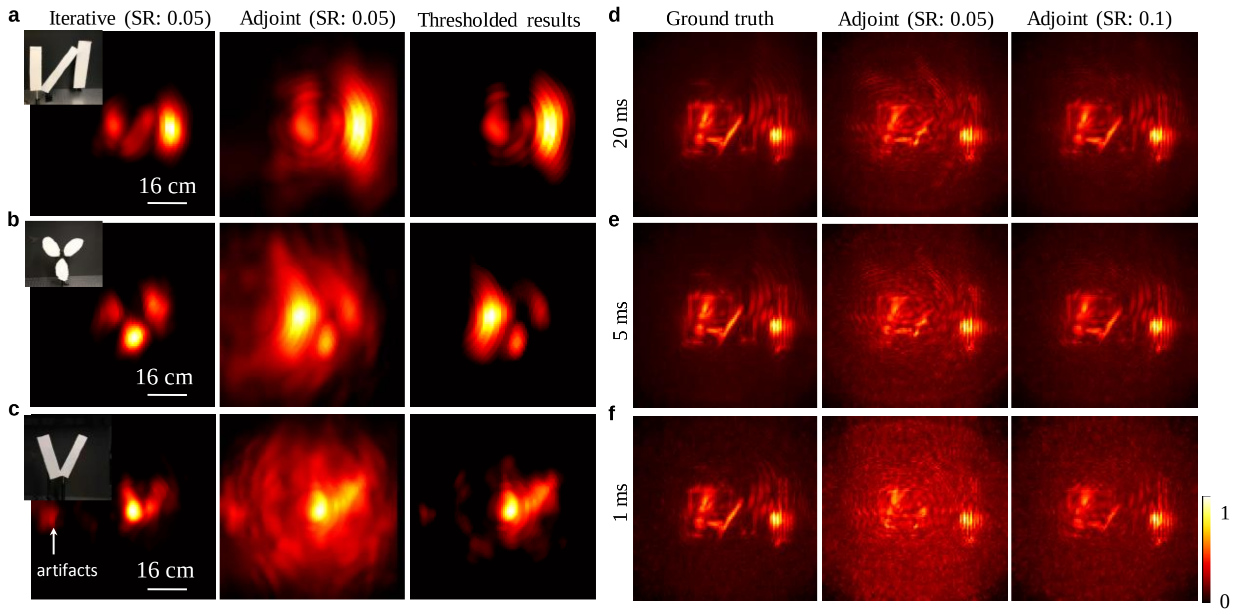

Supplementary Figure 20. NLOS imaging using time-of-flight data recovered by the adjoint methods. a-c Reconstruction of three hidden objects in main Fig. 4 by applying the adjoint method for CLIP recovery. **d-f** Synthetic NLOS imaging for the 'office-scene' dataset, with the exposure time varied from 20 ms to 5 ms and 1 ms from top to bottom. The CLIP sampling ratio is made at SR=0.05 and 0.1 under the tomographic camera embodiments, similar to our current experimental demonstration (SR=0.05). SR: sampling ratio.

Supplementary Note 14. Additional flash LiDAR imaging results

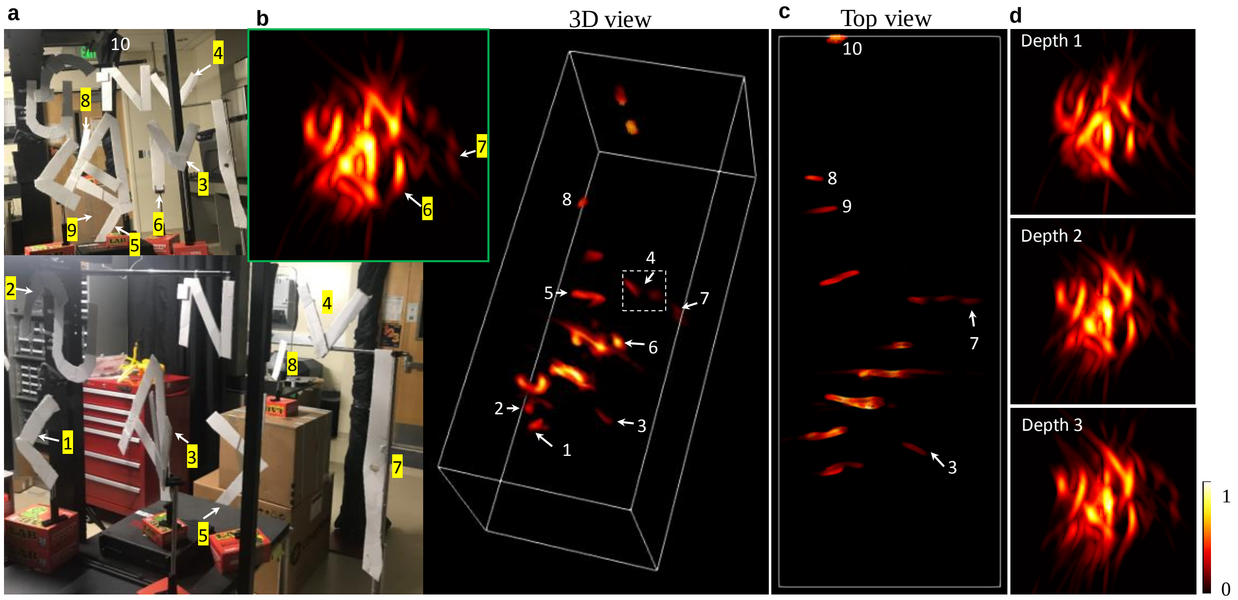

Supplementary Figure 21. Snapshot flash LiDAR imaging over an extended depth range for a cluttered scene. a Reference photographs from the front and side view. **b** A projected 2D LiDAR images of the scene along the depth direction, and a 3D view of the point-cloud representation. **c** Top view of the point cloud. **d** Projected LiDAR images of the 3D scene by refocusing the camera onto a few different focal planes. The complexity of the 3D scene makes it hard to interpret and to compare the LiDAR results with a single 2D photograph. To ease the comparison, we labeled the objects with numbers in both the LiDAR results and photographs. 3D: three-dimensional.

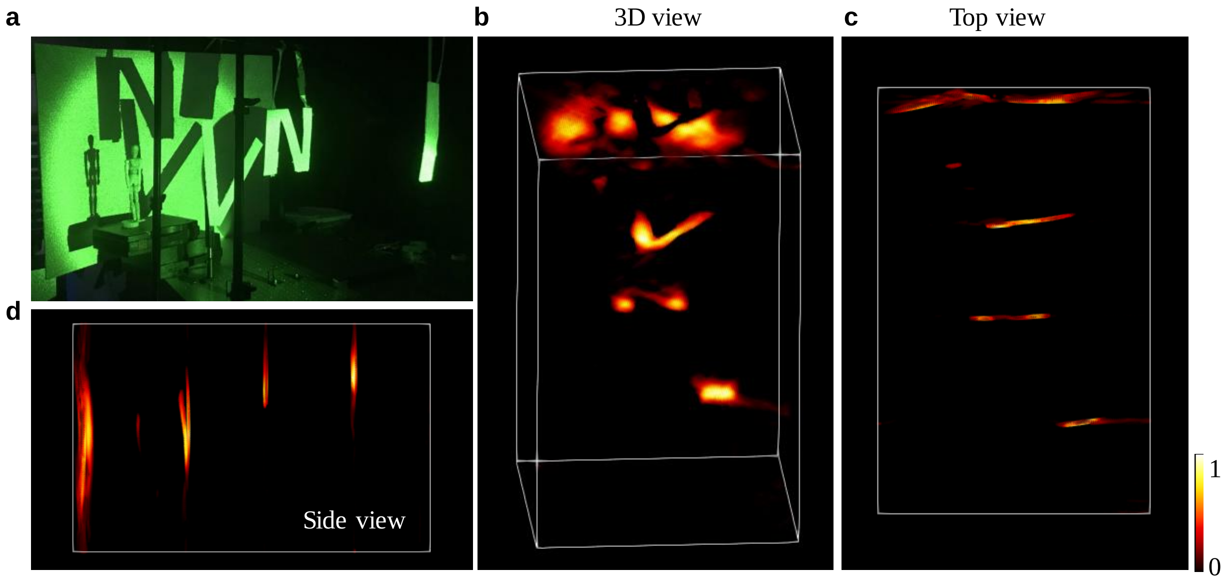

Supplementary Figure 22. Flash LiDAR with a visible 532 nm illumination. To better show the capability of CLIP in recover shadows on a background wall, we changed the illumination to a 532 nm picosecond laser, which allows the ground truth shadow cast by the occluding objects to be captured by a video camera. **a** Reference photographs of the scene with laser illumination. **b-d** Rendering the scene in a 3D view, and from the top and side perspective, respectively. The laser illumination is not uniform across the camera FOV, as revealed by the wall's edges that reproduced the circular illumination pattern. 3D: three-dimensional.

Supplementary References

1. Boykov, Y., Veksler, O. & Zabih, R. Fast Approximate Energy Minimization via Graph Cuts. *IEEE Trans. Pattern Anal. Mach. Intell.* **23**, 18 (2001).
2. Schechner, Y. Y. & Kiryati, N. Depth from Defocus vs. Stereo: How Different Really Are They? *International Journal of Computer Vision* **39**, 141–162 (2000).
3. Levin, A., Fergus, R., Durand, F. & Freeman, W. T. Image and depth from a conventional camera with a coded aperture. *ACM Trans. Graph.* **26**, 70-es (2007).
4. Cohen, N. *et al.* Enhancing the performance of the light field microscope using wavefront coding. *Opt. Express* **22**, 24817 (2014).

5. Marwah, K., Wetzstein, G., Bando, Y. & Raskar, R. Compressive light field photography using overcomplete dictionaries and optimized projections. *ACM Trans. Graph.* **32**, 46:1-46:12 (2013).
6. Cai, Z. *et al.* Lensless light-field imaging through diffuser encoding. *Light Sci Appl* **9**, 143 (2020).
7. Antipa, N., Necula, S., Ng, R. & Waller, L. Single-shot diffuser-encoded light field imaging. in *2016 IEEE International Conference on Computational Photography (ICCP)* 1–11 (IEEE 2016).
8. Levin, A., Hasinoff, S. W., Green, P., Durand, F. & Freeman, W. T. 4D frequency analysis of computational cameras for depth of field extension. *ACM Trans. Graph.* **28**, 97:1-97:14 (2009).
9. Antipa, N. *et al.* DiffuserCam: lensless single-exposure 3D imaging. *Optica*, **5**, 1–9 (2018).
10. Dowski, E. R. & Cathey, W. T. Extended depth of field through wave-front coding. *Appl. Opt* **34**, 1859 (1995).
11. Antipa, N., Oare, P., Bostan, E., Ng, R. & Waller, L. Video from Stills: Lensless Imaging with Rolling Shutter. in *2019 IEEE International Conference on Computational Photography (ICCP)* 1–8 (IEEE 2019).
12. Do, T. T., Gan, L., Nguyen, N. H. & Tran, T. D. Fast and Efficient Compressive Sensing using Structurally Random Matrices. *IEEE Trans. Signal Process.* **60**, 139–154 (2012).
13. Ashok, A. & Neifeld, M. A. Compressive light field imaging. in *Three-Dimensional Imaging, Visualization, and Display 2010 and Display Technologies and Applications for Defense, Security, and Avionics IV* 7690 221–232 (SPIE, 2010).
14. Babacan, S. D. *et al.* Compressive Light Field Sensing. *IEEE Trans. Image Process.* **21**, 4746–4757 (2012).
15. Bastounis, A. & Hansen, A. C. On the absence of the RIP in real-world applications of compressed sensing and the RIP in levels. Preprint at *https://arxiv.org/abs/1411.4449* (2015).
16. Roman, B., Bastounis, A., Adcock, B. & Hansen, A. C. On fundamentals of models and sampling in compressed sensing. Preprint at http://www.damtp.cam.ac.uk/research/afha/people/alexb/papers/CSModels.pdf (2015).
17. Baraniuk, R. G., Cevher, V., Duarte, M. F. & Hegde, C. Model-Based Compressive Sensing. *IEEE Trans. Inform. Theory* **56**, 1982–2001 (2010).
18. Wilburn, B. *et al.* High performance imaging using large camera arrays. *ACM Trans. Graph.* **24**, 765–776 (2005).
19. Liu, X., Bauer, S. & Velten, A. Phasor field diffraction based reconstruction for fast non-line-of-sight imaging systems. *Nat. Commun.* **11**, 1–13 (2020).
20. O’Toole, M., Lindell, D. B. & Wetzstein, G. Confocal non-line-of-sight imaging based on the light-cone transform. *Nature* (2018) doi:10.1038/nature25489.
21. Lindell, D. B., Wetzstein, G. & O’Toole, M. Wave-based non-line-of-sight imaging using fast *f-k* migration. *ACM Trans. Graph.* **38**, 116:1-116:13 (2019).
22. Liu, X. *et al.* Non-line-of-sight imaging using phasor-field virtual wave optics. *Nature* **572**, 620–623 (2019).
