# Supplementary material for: Compact light field photography towards versatile three-dimensional vision
Source: Nat Commun. 2022 Jun 9;13:3333. doi: 10.1038/s41467-022-31087-9 (PMC9184585; doi:10.1038/s41467-022-31087-9)
Supplement: Supplementary file 3 — Description of Additional Supplementary Files [file 41467_2022_31087_MOESM3_ESM.pdf]

Description of Additional Supplementary Files

File Name: Supplementary Movie 1
Description: Dynamic 3D imaging through occlusions Supplementary

File Name: Supplementary Movie 2
Description: Video-rate flash LiDAR imaging of a simple scene

File Name: Supplementary Movie 3
Description: Video-rate flash LiDAR imaging of a complex scene
